# The impact of histone deacetylase inhibition on neurobehavioural outcomes in preclinical models of traumatic and non-traumatic spinal cord injury: a systematic review

**DOI:** 10.3389/fimmu.2025.1690997

**Published:** 2025-11-07

**Authors:** Natalia M. Jagodzinska, Caleb Cole, Jamie Brannigan, Renuka Chintapalli, Benjamin M. Davies, Mark R. Kotter, Oliver D. Mowforth

**Affiliations:** 1 School of Clinical Medicine, University of Cambridge, Cambridge, United Kingdom; 2 Department of Neurology, Mount Sinai Hospital, New York, NY, United States; 3 Division of Academic Neurosurgery, Department of Clinical Neurosciences, University of Cambridge, Cambridge, United Kingdom

**Keywords:** spinal cord injury, histone deacetylase inhibitors, preclinical study, valproate, 4-PBA

## Abstract

**Introduction:**

Spinal cord injury (SCI) is a traumatic injury resulting in significant life-changing disability. Elucidating the molecular processes associated with SCI may help to design novel therapeutics targeted at improving patient outcomes. Current pharmacological candidates include histone deacetylase (HDAC) inhibitors, whose anti-inflammatory properties are postulated to be of value in SCI. The objective was to synthesise the impact of HDAC inhibitors on neurobehavioural outcomes in preclinical studies of traumatic and non-traumatic SCI and to evaluate the suitability of HDAC inhibitors for clinical trials in patients with SCI.

**Methods:**

The review was prospectively registered with PROSPERO (CRD42023477882) and conducted following PRISMA 2020 guidelines. MEDLINE and Embase were searched. Studies of animal models of traumatic or non-traumatic SCI evaluating the effect of HDAC inhibition on neurobehavioural outcomes were eligible for inclusion. Risk of bias was assessed using the SYRCLE checklist. Screening, data-extraction and risk of bias assessments were completed in duplicate.

**Results:**

Of 10,549 studies identified, 42 studies met inclusion criteria. Animal models were rats (n=28), mice (n=13) and rabbits (n=1). SCI models included spinal cord contusion (n=24), epidural compression (n=2), vascular clip compression (n=6), hemisection (n=5), ischaemia/reperfusion injury (n=4) and dorsolateral funiculus crush (n=1). Valproate was the most frequently studied HDAC inhibitor (n=20), followed by 4-phenylbutyrate (4-PBA; n=7) and RGFP966 (n=3). Trichostatin A, tubastatin A, entinostat, PCI-34051, scriptaid, CI-994, TMP269, vorinostat, 3-TYP, SW-100 and ACY1215 were each evaluated in a single study. Three studies used the sirtuin-1 (HDAC class III) inhibitor EX527 administered with an activator molecule: melatonin (n=1), MLN4924 (n=1) and oxymatrine (n=1). Locomotor function was assessed in 98% (41/42) of studies, with improvement in locomotor outcome reported in 73% (30/41). Pain and anxiety were evaluated in one study, in which significant improvement was demonstrated.

**Conclusion:**

HDAC inhibitors are associated with functional motor recovery and improved anxiety and pain scores in preclinical models of SCI. However, the results should be interpreted with caution as risk of bias of included studies was unclear. These results support further investigation of HDAC inhibitors in preclinical studies before translation into clinical trials.

**Systematic Review Registration:**

https://www.crd.york.ac.uk/PROSPERO/, identifier CRD42023477882.

## Introduction

Spinal cord injury (SCI) is a significant public health problem with an estimated 20.6 million individuals affected worldwide and a global incidence of approximately 0.9 million cases each year (1). Currently available treatments have limited efficacy and aim to optimise quality of life rather than reverse the injury (2, 3). Following the acute management phase, care focuses on avoidance of complications and on rehabilitation. This continues for many years after the initial injury. There is therefore an unmet need for better treatments for SCI.

Pathophysiological classifications divide SCI into primary, secondary and chronic phases. Primary injury results from mechanical damage from the initial impact force; secondary injury follows and is divided into acute and subacute phases. The acute phase includes pro-apoptotic signalling leading to cellular dysfunction, death and increased inflammatory cytokine signalling, including tumour necrosis factor alpha (TNFα) and interleukin-1 beta (IL-1β). This promotes macrophage, neutrophil and lymphocyte recruitment, potentiating the inflammatory response. In the subacute phase, cell death follows intracellular Ca2+ dysregulation, glutamate excitotoxicity and free radical release, which hinder neuronal regeneration. Cellular processes in the secondary phase of SCI constitute potential targets for histone deacetylase (HDAC) inhibition (2, 4–6).

HDACs are enzymes that catalyse the removal of acetyl groups from lysine residues of histone and non-histone proteins (7). Removal of acetyl groups from N-terminal tails of histone proteins leads to a more condensed chromatin structure and decreased gene expression (8). HDACs can be divided into four classes (I, II, III, IV) (9–11). Many HDAC inhibitors are pan-inhibitors that target multiple HDACs in class I and II, such as trichostatin A, vorinostat and valproate. More selective HDAC inhibitors include class I inhibitors romidepsin, RGFP966 and entinostat and class III (sirtuin) inhibitors sirtinol, AK-7, splitomicin and nicotinamide (12). Moreover, there are inhibitors that are highly selective for specific HDACs, for example HDAC6-selective inhibitors SW-100 and tubastatin A.

Multiple studies have demonstrated the potential of HDAC inhibitors to interact with molecular pathways important in the mechanisms of SCI (6, 13–17). For example, increased HDAC activity has been detected in nuclear extracts from peripheral blood mononuclear cells after SCI (13) and the neuroprotective properties of HDAC inhibitors have been demonstrated in a mouse model of traumatic brain injury, with increased preservation of myelinated axons and improved neuronal conduction (17). Moreover, in lipopolysaccharide-stimulated macrophages, trichostatin A has been found to reduce the expression of pro-inflammatory cytokines IL-6, TNF-α and IL-1β and increase expression of the immunosuppressive cytokine IL-10 (14). The anti-inflammatory and neuroprotective effects of pan-HDAC inhibitors such as trichostatin A, givinostat, and scriptaid have also been demonstrated across *in vitro*, animal and human studies (6, 14–16).

HDAC inhibitors therefore appear to be promising candidates for adjuvant treatment in SCI. The aim of this systematic review was to study the impact of HDAC inhibitors on neurobehavioural outcomes in preclinical studies of traumatic and non-traumatic SCI and assess potential suitability for clinical trials in SCI patients.

## Methods

### Study design

The systematic review was prospectively registered with PROSPERO (CRD42023477882) and conducted adhering to Preferred Reporting Items for Systematic Reviews and Meta-Analysis (PRISMA 2020) guidelines (18).

#### Eligibility criteria

##### Inclusion criteria

- Animal study
- English language
- Spinal cord injury
- Use of any HDAC inhibitor
- Assessment of any neurobehavioural outcome

##### Exclusion criteria

- Review or meta-analysis
- Editorial
- Letter
- Correction
- Conference abstract
- Full text unavailable

Additional details on the inclusion/exclusion criteria are presented in **Supplementary Table 1**.

### Information sources

MEDLINE and Embase were searched from inception to 14^th^ April 2025. MEDLINE and Embase searches were performed using the Ovid platform (Ovid Technologies, New York, USA).

### Search strategy

Initial scoping searches were performed to refine the review question. The final search strategy was developed over several iterations to maximise the sensitivity (**Supplementary Table 2**). Search sensitivity was assessed using a list of eight articles known to meet inclusion criteria, with all studies successfully captured.

### Selection process

Deduplication was performed using EndNote 21.5 (Clarivate, Philadelphia, United States). Before title and abstract screening, pilot screening of 100 studies was conducted to ensure concordance between screeners. Title and abstract screening was completed by two blinded researchers (NJ, CC) using Rayyan (Rayyan Systems, Cambridge, United States). Disagreements were resolved by discussion. Full text screening was conducted in duplicate by two blinded researchers (NJ, CC). Reasons for exclusions of full-texts are presented in **Supplementary Table 3**.

### Data collection

Data extraction was performed in duplicate by two authors (NJ and CC) in Excel (Microsoft, Washington, United States) using a piloted extraction table.

### Data items

Extracted data points included: author, year, study location, study characteristics, sample characteristics, injury model, intervention, neurobehavioural outcomes, time of assessment, relevant statistical analysis and key findings (**Supplementary Table 4**). We included studies that assessed neurobehavioural outcomes, which are defined as outcomes assessing motor and/or sensory function. Neurobehavioural outcomes of interest included, but were not limited to, locomotor function measured using the Basso, Beattie and Bresnahan (BBB) locomotor scale or the Basso Mouse Scale (BMS), forelimb grip strength and assessments of pain and anxiety. We excluded studies which exclusively assessed non-neurobehavioural outcomes such as electrophysiological measures, autonomic function and histological analysis.

### Risk of bias assessment

To assess the risk of bias of included studies, the Systematic Review Centre for Laboratory Animal Experimentation (SYRCLE) checklist was used (19). The assessment was conducted in duplicate by two blinded and independent researchers (**Supplementary Table 5**). All disagreements were resolved through discussion.

### Synthesis methods

Meta-analysis was not feasible due to the heterogeneity in SCI model design, HDAC therapy administration and outcome measurements. Therefore, a narrative synthesis following the Synthesis Without Meta-analysis (SWiM) reporting guidelines was conducted (20). The SWiM checklist is provided in **Supplementary Table 6**.

Neurobehavioural outcomes were grouped into locomotor, pain and anxiety. For each included study, the differences between the intervention and control group were reported, including statistical tests where available. Due to the diversity of data, the results were transformed into a standardised metric of direction of effect (improvement/deterioration/no effect or conflicting findings) presented in the form of a table and harvest plots (21–23).

## Results

### Study selection

**Figure 1:**
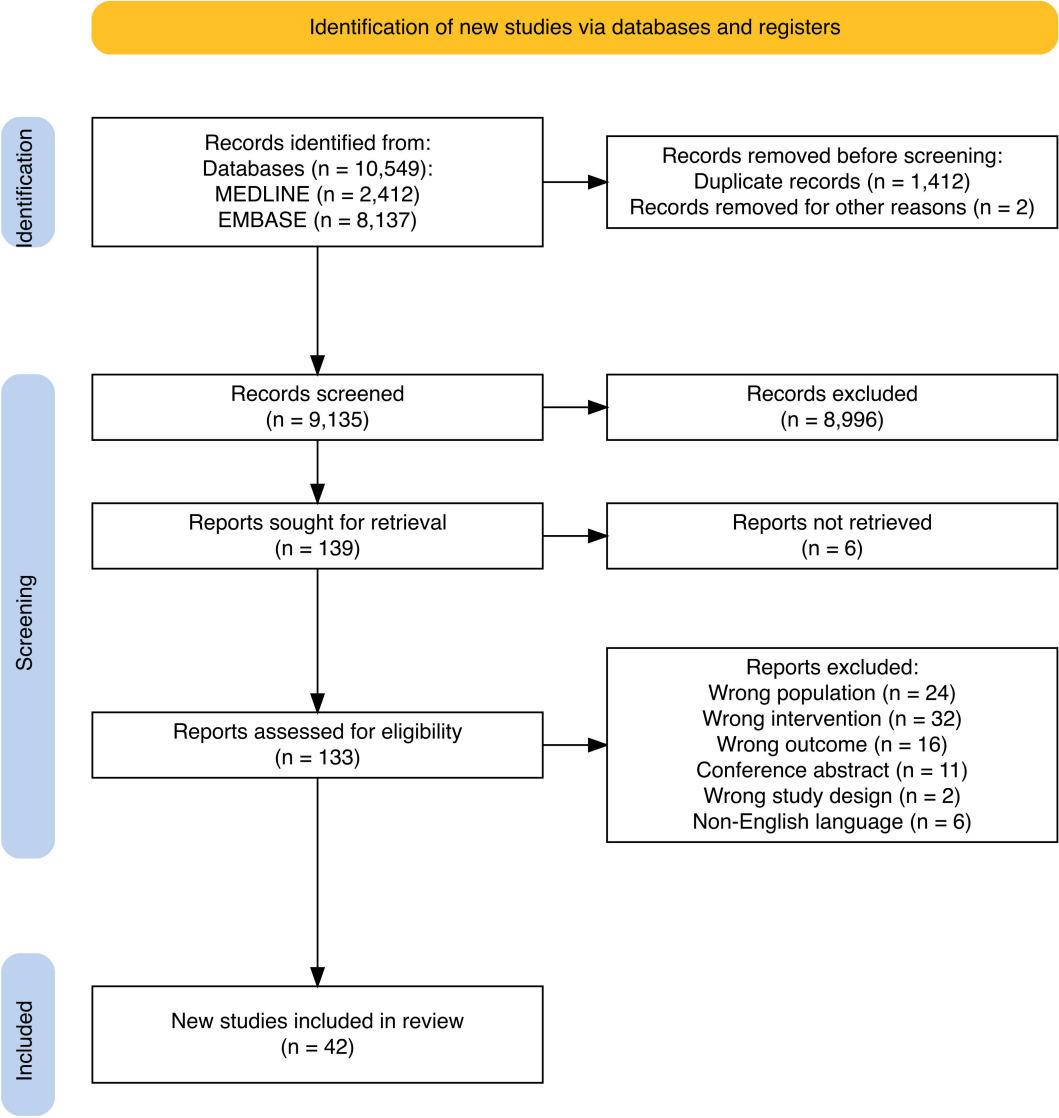
PRISMA flow diagram of study selection (73). After deduplication, 1412 duplicates were removed, leaving 9137 unique studies. Following title and abstract screening, 8996 studies were removed. The remaining 139 studies were included in full text screening. Full texts were not available for 6 studies. A further 91 studies that did not meet eligibility criteria were excluded.

A total of 10,549 records were identified from database searching; 42 studies were included in the final review (**Figure 1**).

### Study characteristics

**Figure 2:**
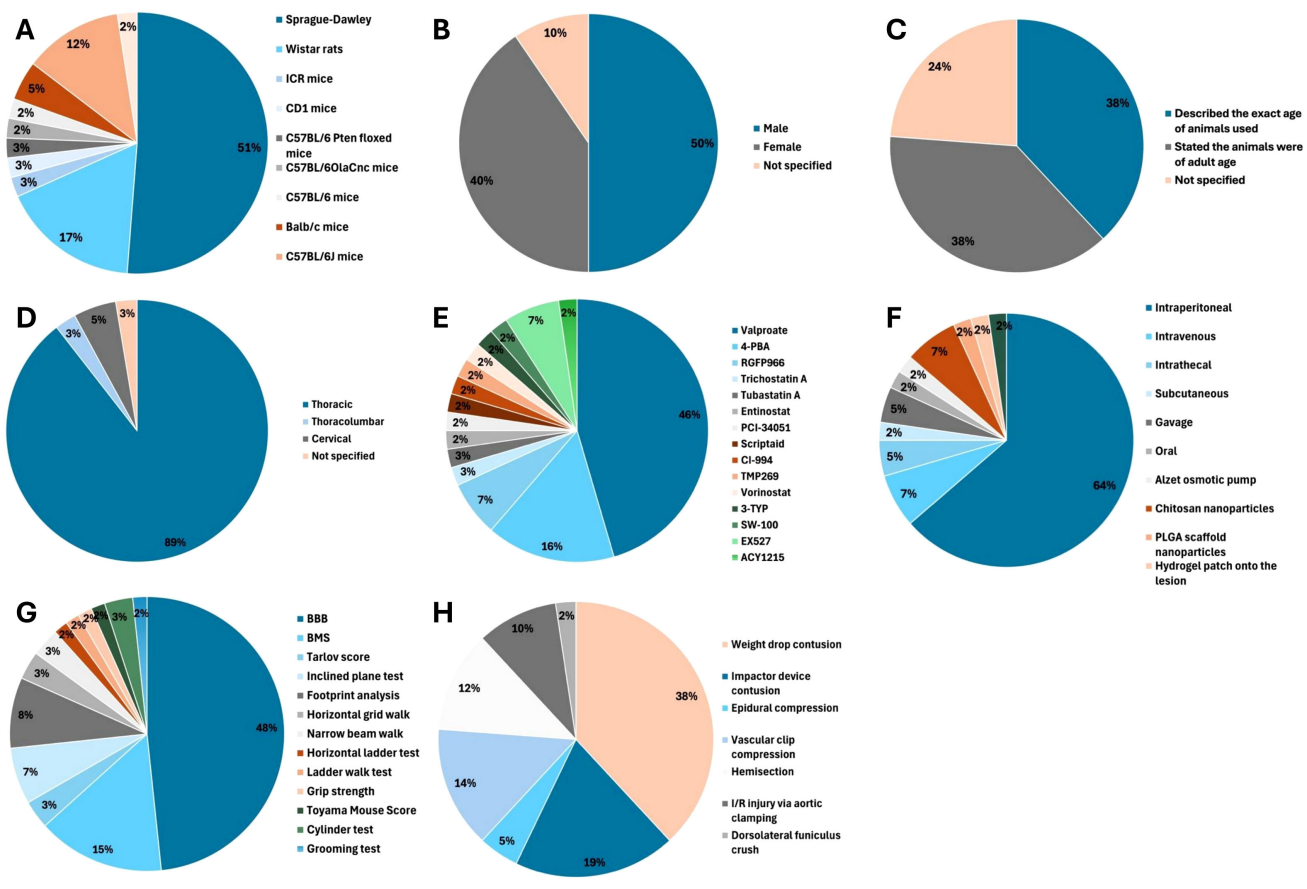
Of 42 included studies, 28 (67%) used rat models (6, 24–47), 13 (31%) used mice (51–59, 61–64) and 1 (2%) study used Japanese white rabbits (60). **(A)** Animal model. **(B)** Sex. **(C)** Age. **(D)** Level of spinal cord injury. **(E)** HDAC inhibitor used. **(F)** Mode of HDAC inhibitor administration. **(G)** Locomotor function test. **(H)** SCI model.

The majority of studies (67%, 28/42) used rat models of SCI (6, 24–50) with Sprague-Dawley rats being most frequently used (**Figure 2** (24–33, 37, 40–49);. Male animals were used in 50% (21/42) of studies (6, 25, 27, 31, 33, 38–43, 45, 47, 48, 50–56), female animals in 40% (17/42) of studies (24, 26, 28, 29, 32, 34–37, 44, 46, 49, 57–61) and 10% (4/42) of studies did not report the sex of animals used (30, 62–64). Most studies (76%, 32/42) reported the age of animals used (6, 25, 28, 29, 31–33, 36–38, 40–47, 49–59, 61, 63, 64). The majority (81%, 34/42) of studies investigated SCI at the thoracic level (6, 25–32, 34, 35, 37, 38, 40–46, 49–59, 61, 62, 64).

A total of 15 different HDAC inhibitors were used, with valproate (VPA) the most commonly evaluated (48%, 20/42) (6, 24, 26, 28, 30–32, 34–42, 45, 50, 51, 57). The time of first administration varied from immediately from before SCI (33, 47, 58, 60), to immediately after SCI (28–31, 35, 38, 44, 46, 54) to seven days later (51). Dose, duration of administration and total duration of treatment varied between studies (**Supplementary Table 4**).

### Neurobehavioural outcome assessment

A variety of neurobehavioural outcome measures were used (**Supplementary Table 7**). Locomotor function was evaluated in 41 (98%, 41/42) studies; pain and anxiety were evaluated in one (2%, 1/42) study (56).

Thirteen different scoring systems of locomotor function were used (**Figure 2**). The Basso, Beattie, Bresnahan (BBB) locomotor score was employed in 29 (69%, 29/42) studies (6, 24–47, 49–51, 54) and the Basso Mouse Scale (BMS) was used in 9 (21%, 9/42) studies (52, 53, 55, 57–59, 61, 62, 64).

Assessments of pain were made using the von Frey hairs test (56) and the thermal paw withdrawal latency test (56). Four tests of anxiety were used: the elevated plus maze test (56), the novelty suppressed feeding test (56), the forced swimming test (56) and the open field test (56). The time of neurobehavioural outcome assessment ranged from 15 minutes (56) to 13 weeks after administration of an HDAC inhibitor (51).

### Effect of HDAC inhibition on locomotor function

**Figure 3:**
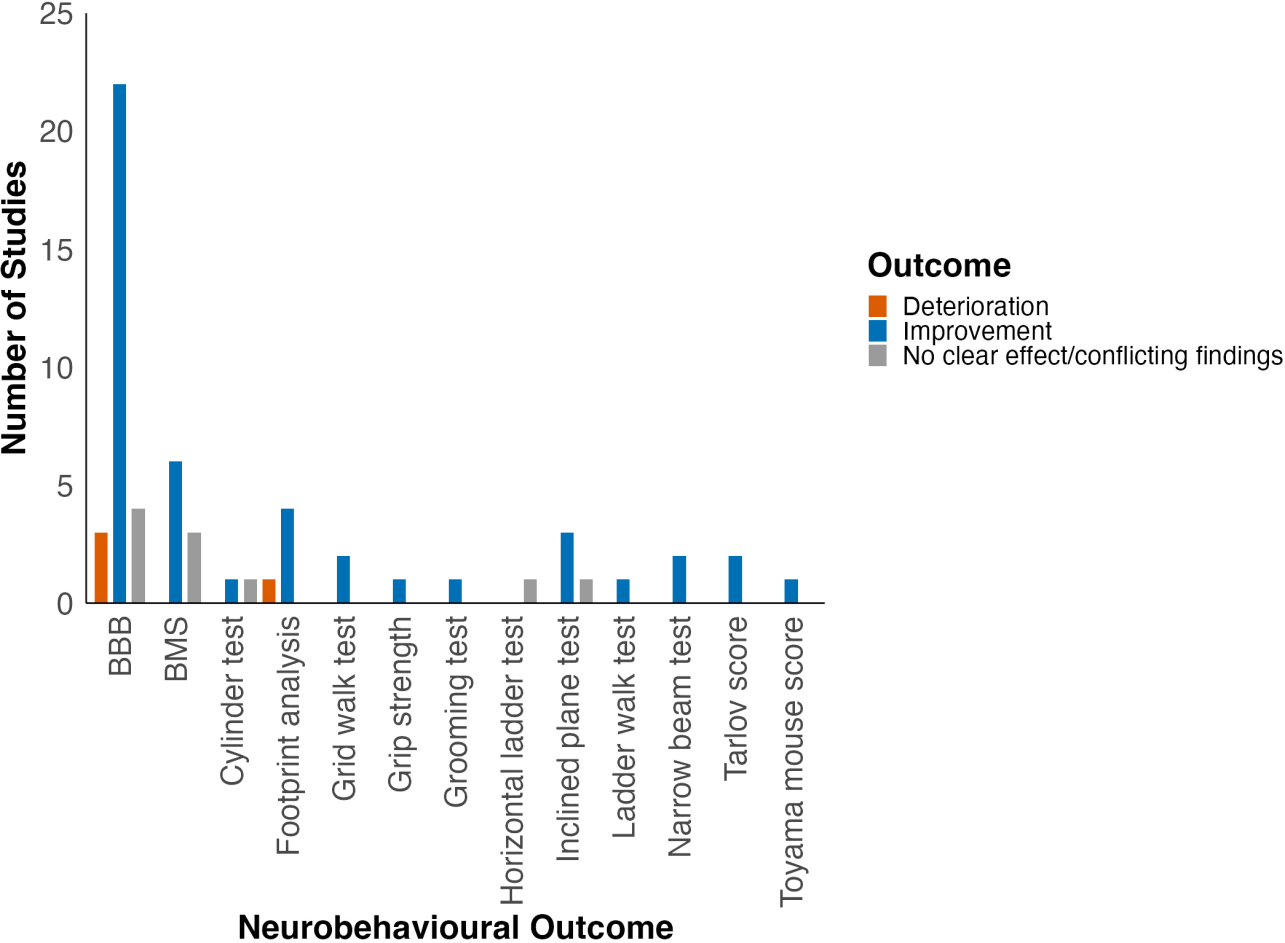
Effect of HDAC inhibition on locomotor function grouped by locomotor outcome. Adapted from Bhatti et al. (2021) (22).

Improvement in locomotor function was observed in studies using BBB, BMS, footprint analysis, grid walk test, grip strength, inclined plane test, narrow beam test, Tarlov score, Toyama mouse score (TMS) and grooming test (**Figure 3**).

**Figure 4:**
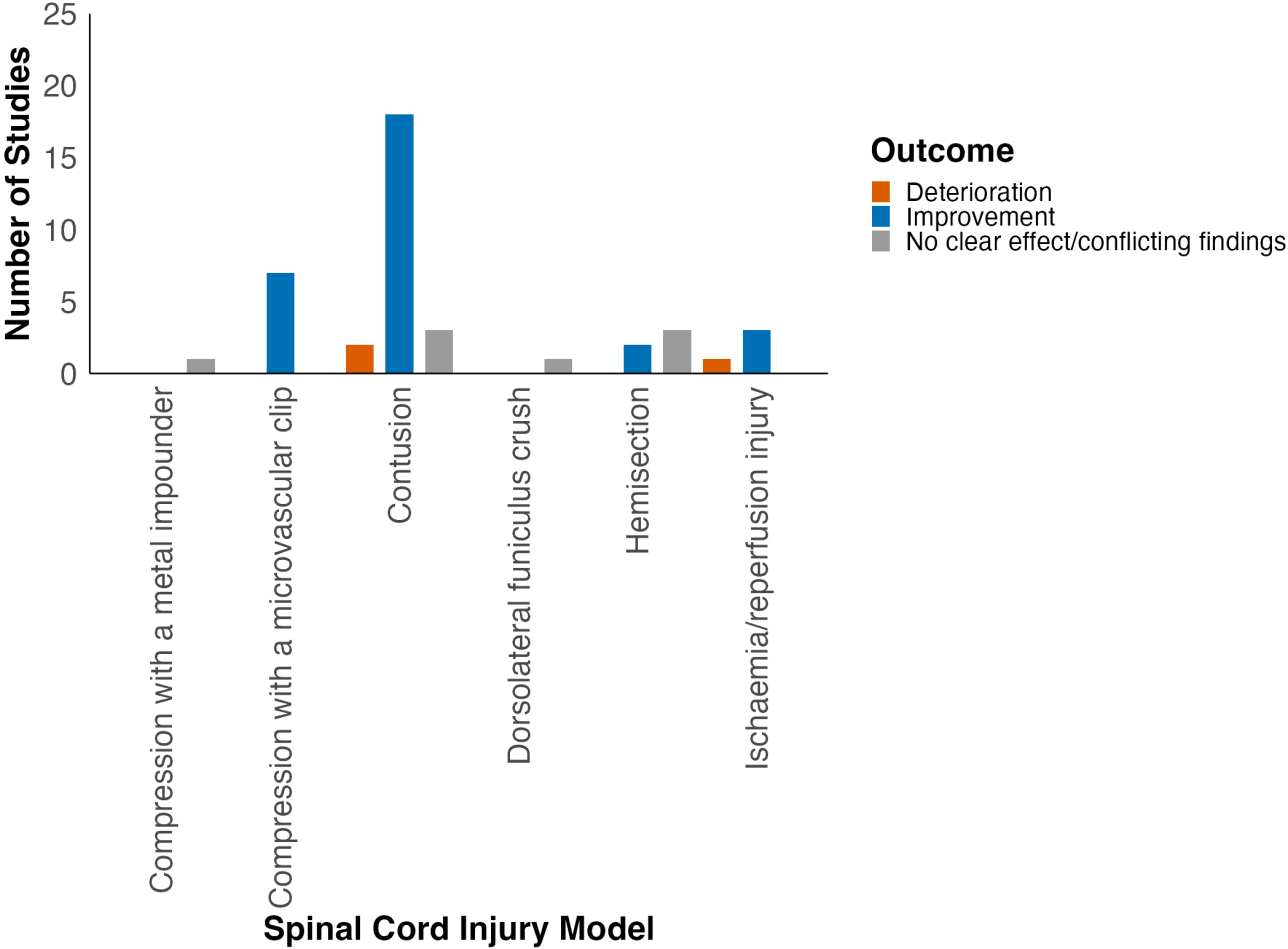
Effect of HDAC inhibition on locomotor function grouped by SCI model type.

Locomotor function was assessed in six different SCI models (**Supplementary Table 8**). Most studies used the contusion model of traumatic SCI (57%, 24/42; **Figure 2**). Improvement in locomotor outcomes appeared most consistent amongst studies using the compression (88%, 7/8) and contusion SCI models (78%, 18/23; **Figure 4**, **Table 1**). Studies using ischaemia/reperfusion injury models also predominantly reported improvement in locomotor outcomes (75%, 3/4). On the contrary, spinal cord hemisection studies predominantly reported no effect of HDAC inhibition on neurobehavioural outcomes.

**Table 1:** Summary of locomotor outcomes in included studies by SCI model.

SCI model	Number of studies	Effect direction
Contusion	23	Improvement: 18 No effect: 4 Deterioration: 1
Compression	8	Improvement: 7 No effect: 1 Deterioration: 0
Ischaemia/Reperfusion injury	4	Improvement: 3 No effect: 0 Deterioration: 1
Spinal cord hemisection	5	Improvement: 2 No effect: 3 Deterioration: 0
Dorsolateral funiculus crush	1	Improvement: 0 No effect: 1 Deterioration: 0

**Figure 5:**
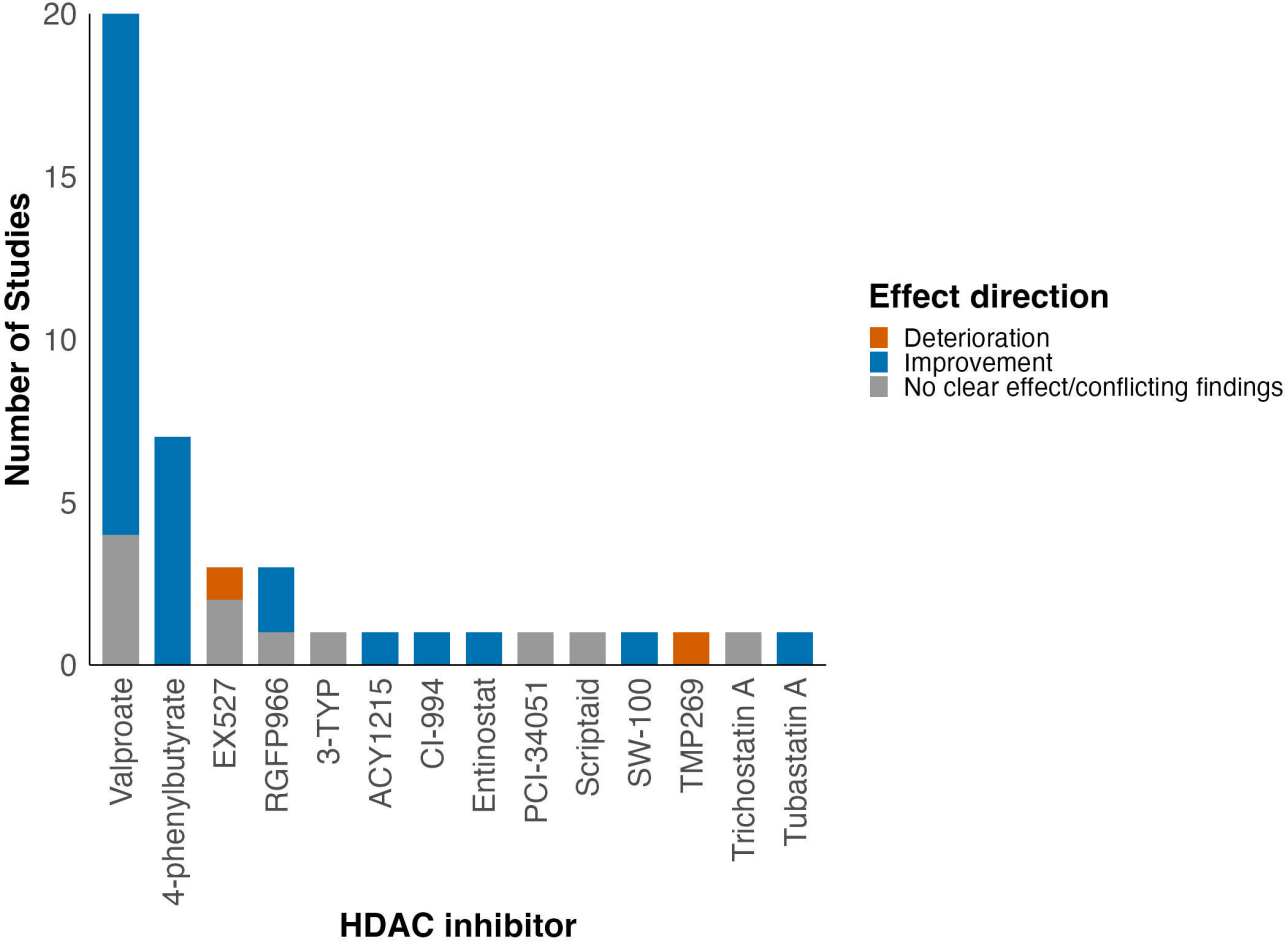
Summary of effect of HDAC inhibition on locomotor function grouped by HDAC inhibitor assessed in each study. A more detailed summary table grouped by HDAC inhibitor class can be found in the **Supplementary Table 9**.

When grouped by HDAC inhibitor class, most studies evaluated pan-HDAC (valproate, 4-PBA, trichostatin A, scriptaid) and class I HDAC inhibitors (RGFP966, entinostat, PCI-34051, CI-994). The most consistent improvement in neurobehavioural outcomes was demonstrated for class IIb HDAC inhibitors (tubastatin A, SW-100, ACY1215; 100%, 3/3), followed by pan-HDAC inhibitors (79%, 23/29) and class I HDAC inhibitors (67%, 4/6; **Table 2**). Moreover, when grouped by HDAC inhibitor used, improvement in locomotor function was seen in studies using 4-PBA, VPA, RGFP966, CI-994, SW-100, entinostat, tubastatin A and ACY1215 (**Figure 5**).

**Table 2:** Summary of locomotor outcomes in included studies by HDAC class. No class IV-selective HDAC inhibitors were used in the included studies.

HDAC inhibitor class	Number of studies	Effect direction
Class I	6	Improvement: 4 No effect: 2 Deterioration: 0
Class IIa	1	Improvement: 0 No effect: 0 Deterioration: 1
Class IIb	3	Improvement: 3 No effect: 0 Deterioration: 0
Class III	4	Improvement: 0 No effect: 3 Deterioration: 1
Pan-HDAC inhibitors*	29	Improvement: 23 No effect: 6 Deterioration: 0

*Pan-HDAC inhibitors: inhibit more than one class of HDACs: valproate (class I and IIa), trichostatin A (class I and II), scriptaid (class I and IIb).

### Valproate

Administration of VPA was associated with improved neurobehavioural outcomes in 80% (16/20) of studies (6, 24, 26, 28, 30–32, 34–37, 39–42, 45). However, four studies reported no significant difference in functional outcomes between treatment and control groups at any time point (38, 50, 51, 57).

Abetmatsu et al. (2010) assessed VPA treatment alone and in combination with a neural stem cell transplant and identified conflicting findings (51). Mice that received neural stem cell treatment alone and those that received it in combination with VPA showed significant improvement in locomotor function compared to untreated SCI mice (51). However, mice treated with VPA alone showed no improvement in locomotor function (BBB score) compared to untreated SCI mice. Conflicting results were also seen when assessing the combinatorial efficacy of VPA delivered on chitosan nanoparticles compared to VPA administered alone. In one study, after treatment with VPA on chitosan nanoparticles, BBB scores were significantly improved (40). However, VPA or chitosan nanoparticle treatment alone resulted in no improvement in BBB score at any time point assessed (40). In the second study, BBB scores were significantly increased in the VPA and chitosan nanoparticles group and in the VPA-only group (41).

### 4-phenylbutyrate

Improvement in neurobehavioural outcomes was observed in all seven studies using 4-PBA (29, 33, 43, 44, 48, 53, 60). For example, 4-PBA treatment was associated with significantly improved locomotor function (BBB score) in rats after ischaemia/reperfusion SCI both alone and when used simultaneously with xenon postconditioning (33). Moreover, Zhou et al. (2016) reported 4-PBA treatment administered immediately after SCI followed by daily administration for two weeks significantly improved BBB score 6–14 days after injury in rats following vascular clip compression SCI (44). Furthermore, Lanza et al. (2019) reported a significant improvement in BMS score in mice treated with 4-PBA following SCI (53). Additionally, He et al. (2017) demonstrated the maximum angle at which an animal can maintain its grip on an inclined plane was significantly increased in diabetic rats with SCI who were treated with 4-PBA (29).

### Class I HDAC inhibitors (RGFP966, entinostat, CI-994, PCI-34051)

RGFP966 was used in three studies with two (67%) demonstrating improvement in locomotor scores including BBB, BMS and TMS following contusional SCI in mice and rats compared to untreated SCI animals (25, 59). Another study by Sanchez et al. (2018) used a hemisection SCI model and showed no difference in hindlimb movements (BMS scores) between mice treated with RGFP966 and the untreated SCI group (61).

One study of entinostat demonstrated improvement in locomotor function assessed using BMS score and forelimb grip strength in mice following a compression SCI (52). Another study in which CI-994 was administered once daily for 14 days after induction of a dorsal hemisection SCI demonstrated overall improvement in several measures of locomotor function including BMS score, narrow beam walk test, horizontal grid walk test and ladder walk test in treated mice (64).

In contrast, Hendrix et al. (2020) administered PCI-34051 to mice following spinal cord hemisection and found no effect of treatment on locomotor recovery assessed using the BMS score (57).

### Class IIb inhibitors (tubastatin A, SW-100, ACY1215)

Zheng et al. (2020) demonstrated improvement in BMS score and footprint patterns in mice treated with tubastatin A compared to untreated mice, suggesting improvement in hindlimb weakness after SCI at 28 days after injury (55). Another study evaluating SW-100 demonstrated improved locomotor function (BMS score) after administration of SW-100 alongside miR-34a-5p inhibitor delivered in exosomes following a contusion SCI (62). A study by Dai et al. (2024) found that treatment with ACY1215 led to significant improvement in locomotor scores (BBB) in SCI rats compared to untreated SCI rats (49).

### Class III inhibitors (EX527, 3-TYP)

All studies using EX527 used a sirtuin activator compound to investigate whether activation of the sirtuin pathway may have beneficial effects in improving the locomotor function following SCI. In all three studies, addition of EX527 which is a sirtuin 1 inhibitor led to reduction in BBB scores reflecting poorer motor function compared to the SCI + sirtuin activator alone group (27, 46, 47).

In a study assessing 3-TYP in mice, BMS score in the treated group was not significantly different from that of the untreated-SCI mice (58).

### Other HDAC inhibitors (trichostatin A, scriptaid, TMP269)

Trichostatin A (class I and II HDAC inhibitor) treatment was associated with age-dependent opposite effects in mice after SCI: in older animals it was associated with significantly higher foot slip cumulative error score in a horizontal ladder test corresponding to poor locomotor function, whilst the opposite was observed in young mice (63).

Studies using scriptaid (class I and IIb HDAC inhibitor) in mice following hemisection SCI demonstrated no difference in functional outcomes between treated and control groups (61). However, one study of mice treated with TMP269 (class IIa HDAC inhibitor) showed reduced hindlimb movement compared with vehicle-treated group that was maintained for up to 6 weeks after injury (54).

### Effect of HDAC inhibition on pain and anxiety

One study assessed the effects of vorinostat on pain and anxiety following contusion SCI. It used two outcome measures for pain: the von Frey filament test and the thermal paw withdrawal latency test and four outcome measures for anxiety: the elevated plus maze test, the novelty suppressed feeding test, the forced swimming test and the open field test. Both tests for pain demonstrated significant improvement after HDAC inhibitor treatment. In the assessment of anxiety behaviours, none of the tests used reached statistical significance but they all demonstrated direction of effect favouring vorinostat treatment (56).

### Risk of bias assessments

Only one (2%, 1/42) study adequately generated and applied the allocation sequence. A total of 62% (26/42) of studies reported baseline characteristics. The allocation sequence was adequately concealed in 5% (2/42) of studies. None of the included studies reported whether animals were randomly housed during the experiment. One (2%, 1/42) study reported the investigators were blinded. None of the studies reported if the animals were selected at random for the outcome assessment. The outcome assessor was blinded in 69% (29/42) of studies. Incomplete outcome data were adequately addressed in 74% (31/42) of studies. Selective outcome reporting was noted in 12% (5/42) of studies. A total of 19% (8/42) studies chose insufficient control groups which may have contributed to selection bias. Overall, the risk of bias was therefore unclear (**Supplementary Table 5**).

## Discussion

### Summary of main findings

The aim of this review was to synthesise the evidence on the effect of HDAC inhibitors on neurobehavioural outcomes in preclinical models of SCI. We found that the majority of class I, class IIb and pan-HDAC inhibitors were associated with beneficial effects on neurobehavioural outcomes in animal models of SCI. The only exceptions were trichostatin A, scriptaid and PCI-34051 which had unclear effect on locomotor function, and TMP269 which was associated with poorer functional recovery after SCI. Class III inhibitors appeared to have no effect or be associated with poorer locomotor function following SCI.

### Differences in HDAC inhibitor mechanisms of action and heterogeneity in neurobehavioural outcomes

Class I and IIb HDAC inhibitors appeared to exhibit the most consistent neuroprotective effects following SCI. Their mechanisms of action are similar and include reduction of inflammatory and apoptotic signalling and promotion of autophagy through increased microtubular transport and decreased neuronal endoplasmic reticular stress at the site of SCI (**Supplementary Table 10**) 6, 36, 60).

For example, Zheng et al. (2020) studied class IIb HDAC inhibitors and found that HDAC6 expression increases at the SCI site and is associated with impaired autophagy and increased neuronal apoptosis. Inhibition of HDAC6 appears to increase tubulin acetylation, supporting motor protein recruitment and retrograde transport in neurones. This is particularly important for autophagy (55), with failure associated with increased neuronal apoptosis. Class IIb HDAC inhibitors may therefore promote neuronal survival following SCI (65).

Class I HDAC inhibitors appear to have significant anti-inflammatory effects. For example, HDAC3 was found to significantly contribute to SCI pathogenesis, particularly through its role in activation of the inflammatory response (6). Inhibiting class I HDACs, especially HDAC3, was found to reduce microglial activation and restrict production of pro-inflammatory cytokines (TNF-α, IL-1β, IL-6) (6). An increase in sirtuin 1 expression is associated with HDAC3 inhibition (25). This is consistent with the findings from our review that inhibition of class III HDACs (of which sirtuin 1 is an example) had either no effect or was associated with poorer functional recovery following SCI (25, 66). Moreover, Sanchez et al. (2018) demonstrated that HDAC3 inhibition promotes a shift from the M1-like macrophage phenotype to anti-inflammatory, pro-regenerative M2-like phenotype further supporting the role of class I HDACs in inflammation following SCI (61).

In addition, valproate, which predominantly inhibits class I HDACs, has been associated with increased expression of BDNF and GDNF neurotrophic factors *in vitro*, promoting neuroregeneration and counterbalancing the inhibitory environment for neuronal growth driven by Nogo-A (34, 67).

Therefore, HDAC3 (class I HDAC) and HDAC6 (class IIb HDAC) appear particularly promising targets in the context of SCI treatment and more studies of selective HDAC3 (e.g. RGFP966) and HDAC6 (e.g. SW-100, ACY1215) inhibitors are needed to elucidate their mechanism and efficacy in improving neurological function following SCI.

Interestingly, HDAC5 inhibition seems to have an important role in regulation of pain following SCI. The mechanism behind this effect may be related to reduction in Nav1.7 channel expression following targeted protein degradation (56). As Nav1.7 channels are known to play an important role in nociception, HDAC5 inhibition represents a promising research avenue, even beyond spinal cord injury (56).

In contrast, class IIa HDAC inhibition (e.g. TMP269) appears to promote inflammation at the injury site, an effect associated with a shift in macrophage polarisation towards the M1-type (54). This may explain why the HDAC inhibitors which target both class I and class IIa/IIb HDACs (VPA, trichostatin A) do not always appear to be associated with definitive or significant improvement in functional outcomes following SCI in included studies.

Similarly to class IIa inhibition, class III HDAC inhibition increases oxidative damage in neurones, promotes apoptosis and reduces autophagy following SCI. However, activators of class III HDACs (melatonin, MLN4924, oxymatrine) improve functional outcomes following SCI (25, 27, 46, 47, 68).

### Opportunities for translational clinical trials

To date, no clinical trials assessing effects of HDAC inhibitors on functional outcomes following SCI have been conducted. The only clinical trial assessing an HDAC inhibitor in patients after SCI was conducted by Drewes et al. (1994) and assessed the effects of valproate treatment on chronic central pain after SCI (69).

Overall, class I and IIb HDAC inhibitors appear to have beneficial effects on locomotor function, pain and anxiety after SCI in animals suggesting that HDAC inhibition may have potential to improve patient outcomes in clinical trials. The review identified VPA and 4-PBA as having the most favourable neurobehavioural outcomes amongst the reviewed studies with 80% of studies using VPA and 100% of studies using 4-PBA reporting improvement in neurobehavioural outcomes making them the best candidates for further studies. However, due to the high heterogeneity and unclear risk of bias observed in those studies, more preclinical evidence is required. Additionally, it may be useful to assess the effects of other FDA-approved HDAC inhibitors (**Supplementary Table 11**) on neurobehavioural outcomes in animal models of SCI, given that the toxicity profiles of these drugs are already well-understood, which may simplify translation of preclinical evidence into future clinical trials.

### Limitations

Firstly, limited reporting, scored using the SYRCLE risk of bias assessments, affects certainty about the quality of the results of included studies. This limits certainty of conclusions. Selection bias, performance bias and detection bias related questions, including adequate generation of the allocation sequence, baseline characteristics, blinding of caregivers and investigators and details on housing of animals were poorly reported. Therefore, risk of bias was unclear. Lack of adequate reporting appears to be an issue with many preclinical studies and is thought to be related to a historical lack of strict reporting requirements for animal studies. A systematic review by Bhatti et al. (2021) advocated for the widespread use of the Animal Research: Reporting of *In Vivo* Experiments (ARRIVE) guidelines to improve the quality of evidence from preclinical studies (22, 70).

In addition, the included studies are highly heterogenous. There is significant variability in SCI models used, timing and route of administration of HDAC inhibitors. There were also significant differences in the severity and mechanism of SCI between models. For example, it is recognised that in the contusion model there may be axonal sparing, which can be falsely interpreted as neuronal regeneration at the lesion site. On the other hand, hemisectional SCI should not cause axonal sparing at the lesion site and any observed regeneration can be more confidently attributed to effects of the studied treatment (71).

The selectivity of HDAC inhibitors differs amongst the drugs included in the review. The current evidence suggests that the main HDACs that should be targeted in SCI are HDAC3 and HDAC6. The most studied inhibitor in context of SCI was valproate. This targets both class I and class II HDACs, which may explain contradictory results in some of the studies.

Furthermore, certain methods of HDAC inhibitor delivery may be difficult to implement in the clinical environment. For example, intrathecal delivery is technically challenging and carries higher risks of infection and neurological toxicity compared to other methods of administration (72).

### Future directions

To generate more robust and translatable evidence, there is a need for larger, well-reported preclinical studies of HDAC inhibitors. Secondly, standardised SCI models for testing HDAC inhibitors may help to alleviate heterogeneity observed within each HDAC inhibitor group studied. Moreover, standardisation of the dose, time and route of administration is also important. In addition, there is a need for more mechanistic studies of HDAC3 and HDAC6 inhibitors which have significant potential for SCI treatment and are limited by few studies of their neurobehavioural effects and mechanism of action compared to 4-PBA and valproate.

## Conclusion

Class I and class IIb HDAC inhibitors are associated with functional locomotor recovery and improved pain and anxiety scores in preclinical models of SCI. By contrast, class III HDAC inhibitors and class IIa HDAC inhibitors are associated with either no effect or deterioration in functional recovery after SCI. However, due to unclear risk of bias in all included studies and high heterogeneity amongst study characteristics, the results should be interpreted with caution. Nevertheless, these findings may be helpful in recognising promising targets for future translational research, including HDAC3 and HDAC6.

## Data Availability

The original contributions presented in the study are included in the article/**Supplementary Material**. Further inquiries can be directed to the corresponding author.
