# Supplementary material for: The impact of histone deacetylase inhibition on neurobehavioural outcomes in preclinical models of traumatic and non-traumatic spinal cord injury: a systematic review
Source: Front Immunol. 2025 Nov 7;16:1690997. doi: 10.3389/fimmu.2025.1690997 (PMC12634382; doi:10.3389/fimmu.2025.1690997)
Supplement: Supplementary file 1 [file DataSheet1.docx]

**Table 1: Inclusion and exclusion criteria**

For a study to be included it must have met all the inclusion criteria and none of the exclusion criteria.

| **Exclusion reason** | **Type in Rayyan reason box** | **Exclusion hierarchy** |
| --- | --- | --- |
|  | | |
| 1. **WRONG PATIENT POPULATION** | | |
| No spinal cord injury animal model used | Wrong population | 1 |
| Clinical study | Clinical | 1 |
| Human study | Human | 1 |
| Ex vivo | Ex vivo | 1 |
| In vitro | In vitro | 1 |
| In silico | In silico | 1 |
| 1. **WRONG INTERVENTION OR OUTCOME** | | |
| No HDAC inhibitor given | No HDAC inhibitor | 2 |
| No neurobehavioural outcome | No neurobehavioural outcome | 2 |
|  | | |
| 1. **WRONG STUDY DESIGN** | | |
| Systematic Review | Systematic Review | 3 |
| Narrative Review | Narrative Review | 3 |
| Other Review Article | Other Review | 3 |
| Meta-analysis | Meta-analysis | 3 |
| Case Report | Case Report | 3 |
| Survey | Survey | 3 |
| Letter | Letter | 3 |
| Editorial | Editorial | 3 |
| Opinion Article | Opinion | 3 |
| Correction | Correction | 3 |
| Conference Abstract | Conference | 3 |
| Audit | Audit | 3 |
| Guideline | Guideline | 3 |
| Lecture | Lecture | 3 |
| Commentary | Commentary | 3 |
| Perspective | Perspective | 3 |
| Research Highlight | Research Highlight | 3 |
| Correspondence | Correspondence | 3 |
| Feature | Feature | 3 |
| News article | News | 3 |
| Message from the editor | Message from the editor | 3 |
| Learning module | Learning module | 3 |
| Fact sheet | Fact sheet | 3 |
| Insight | Insight | 3 |
| Preview | Preview | 3 |
| Meeting report | Report | 3 |
| Historical note | Historical note | 3 |
| Book chapter | Chapter | 3 |
| Pharmacological pipeline | Pipeline | 3 |
| Prospect | Prospect | 3 |
| Proceedings | Proceedings | 3 |
|  | | |
| 1. **WRONG LANGUAGE** | | |
| Non-English Language | Language | 4 |
|  | | |
| 1. **FULL TEXT MISSING** | | |
| Full-text unavailable | Full text | 5 |

**Table 2: Search strategy**

**Database:** Ovid MEDLINE(R) and Epub Ahead of Print, In-Process, In-Data-Review & Other Non-Indexed Citations, Daily and Versions <1946 to January 08, 2024>

| **#** | **Search terms** | **Number of articles found** |
| --- | --- | --- |
| **1** | exp Histone Deacetylase Inhibitors/ or exp Histone Deacetylases/ or exp Vorinostat/ or exp Panobinostat/ or exp Depsipeptides/ or exp Valproic Acid/ or exp Butyric Acid/ or exp Phenylbutyrates/ or (HDAC inhibit* or histone deacetylase inhibit* or HDACi* or HDI or HDAC immunotherap* or histone deacetylase immunotherap* or HDACi immunotherap* or HDI immunotherap* or vorinostat or SAHA or belinostat or PXD101 or Panobinostat or LBH589 or trichostatin or TSA or tubacin or depsipeptide* or apicidine or trapoxin or entinostat or MS-275 or M275 or mocetinostat or valproic acid or valproate or sodium butyrate or phenylbutyr* or phenyl butyr* or givinostat or ITF2357).mp. | **87077** |
| **2** | ((cervical or thoracic or lumbar or spin* or vertebr* or nerve* or neuro* or cord) adj4 (lesion* or contus* or concuss* or compress* or damag* or trauma* or injur* or sever* or transect* or degenerat* or myelinat* or remyelinat* or demyelinat* or lacerat* or myelopath*)).mp. | **474297** |
| **3** | exp Spinal Cord Injuries/ or exp Spinal Diseases/ or exp Spinal Cord Compression/ or exp Nerve Degeneration/ or exp Spinal Cord Vascular Diseases/ or exp Paralysis/ or exp Spine/ or exp Spinal Cord/ | **487734** |
| **4** | 2 or 3 | **818389** |
| **5** | 1 and 4 | **2163** |

**Database:** Embase <1974 to 2024 January 08>

| # | **Search terms** | **Number of articles found** |
| --- | --- | --- |
| 1 | exp histone deacetylase/ or exp histone deacetylase inhibitor/ or exp vorinostat/ or exp belinostat/ or exp panobinostat/ or exp trichostatin A/ or exp tubacin/ or exp depsipeptide/ or exp entinostat/ or exp mocetinostat/ or exp butyric acid/ or exp valproic acid/ or exp 4 phenylbutyric acid/ or exp phenylbutyrate/ or exp phenylbutyric acid/ or exp givinostat/ or (HDAC inhibit* or histone deacetylase inhibit* or HDACi* or HDI or HDAC immunotherap* or histone deacetylase immunotherap* or HDACi immunotherap* or HDI immunotherap* or vorinostat or saha or belinostat or PXD101 or panobinostat or LBH589 or trichostatin or TSA or tubacin or apicidine or depsipeptide or trapoxin or entinostat or MS-275 or M275 or mocetinostat or sodium butyrate or butyric acid or valproic acid or valproate or phenyl butyr* or phenylbutyr* or givinostat or ITF2357).mp. | **230166** |
| 2 | ((cervical or thoracic or lumbar or spin* or vertebr* or nerv* or neuro* or cord) adj4 (lesion* or contus* or concuss* or compress* or damag* or trauma* or injur* or sever* or transect* or degenerat* or myelinat* or remyelinat* or demyelinat* or lacerat* or myelopath*)).mp. | **699933** |
| 3 | exp spine disease/ or exp cervical myelopathy/ or exp spinal cord atrophy/ or exp spinal cord injury/ or exp spinal cord lesion/ or exp spinal cord vascular disease/ or exp spinal paralysis/ or exp nerve degeneration/ or exp spine/ or exp spinal cord/ | **698074** |
| 4 | 2 or 3 | **1125690** |
| 5 | 1 and 4 | **7384** |

**Total Search Results 9,547**

**With Duplicates Removed 8,259**

The search was done on 08.01.2024. Number of citations for each component of the search is shown in bold.

Search was updated on 14.04.2025 and the results between the period of 8^th^ January 2024 and 14^th^ April 2025 are presented below.

**Database:** Ovid MEDLINE(R) and Epub Ahead of Print, In-Process, In-Data-Review & Other Non-Indexed Citations, Daily and Versions <1946 to 11 April 2025>

| **#** | **Search terms** | **Number of articles found** |
| --- | --- | --- |
| **1** | exp Histone Deacetylase Inhibitors/ or exp Histone Deacetylases/ or exp Vorinostat/ or exp Panobinostat/ or exp Depsipeptides/ or exp Valproic Acid/ or exp Butyric Acid/ or exp Phenylbutyrates/ or (HDAC inhibit* or histone deacetylase inhibit* or HDACi* or HDI or HDAC immunotherap* or histone deacetylase immunotherap* or HDACi immunotherap* or HDI immunotherap* or vorinostat or SAHA or belinostat or PXD101 or Panobinostat or LBH589 or trichostatin or TSA or tubacin or depsipeptide* or apicidine or trapoxin or entinostat or MS-275 or M275 or mocetinostat or valproic acid or valproate or sodium butyrate or phenylbutyr* or phenyl butyr* or givinostat or ITF2357).mp. | **93615** |
| **2** | ((cervical or thoracic or lumbar or spin* or vertebr* or nerve* or neuro* or cord) adj4 (lesion* or contus* or concuss* or compress* or damag* or trauma* or injur* or sever* or transect* or degenerat* or myelinat* or remyelinat* or demyelinat* or lacerat* or myelopath*)).mp. | **503625** |
| **3** | exp Spinal Cord Injuries/ or exp Spinal Diseases/ or exp Spinal Cord Compression/ or exp Nerve Degeneration/ or exp Spinal Cord Vascular Diseases/ or exp Paralysis/ or exp Spine/ or exp Spinal Cord/ | **505520** |
| **4** | 2 or 3 | **860040** |
| **5** | 1 and 4 | **2434** |
| **6** | limit 5 to dt=20240108-20250414 | **249** |

**Database:** Embase <1974 to 2025 April>

| # | **Search terms** | **Number of articles found** |
| --- | --- | --- |
| 1 | exp histone deacetylase/ or exp histone deacetylase inhibitor/ or exp vorinostat/ or exp belinostat/ or exp panobinostat/ or exp trichostatin A/ or exp tubacin/ or exp depsipeptide/ or exp entinostat/ or exp mocetinostat/ or exp butyric acid/ or exp valproic acid/ or exp 4 phenylbutyric acid/ or exp phenylbutyrate/ or exp phenylbutyric acid/ or exp givinostat/ or (HDAC inhibit* or histone deacetylase inhibit* or HDACi* or HDI or HDAC immunotherap* or histone deacetylase immunotherap* or HDACi immunotherap* or HDI immunotherap* or vorinostat or saha or belinostat or PXD101 or panobinostat or LBH589 or trichostatin or TSA or tubacin or apicidine or depsipeptide or trapoxin or entinostat or MS-275 or M275 or mocetinostat or sodium butyrate or butyric acid or valproic acid or valproate or phenyl butyr* or phenylbutyr* or givinostat or ITF2357).mp. | **245473** |
| 2 | ((cervical or thoracic or lumbar or spin* or vertebr* or nerv* or neuro* or cord) adj4 (lesion* or contus* or concuss* or compress* or damag* or trauma* or injur* or sever* or transect* or degenerat* or myelinat* or remyelinat* or demyelinat* or lacerat* or myelopath*)).mp. | **747213** |
| 3 | exp spine disease/ or exp cervical myelopathy/ or exp spinal cord atrophy/ or exp spinal cord injury/ or exp spinal cord lesion/ or exp spinal cord vascular disease/ or exp spinal paralysis/ or exp nerve degeneration/ or exp spine/ or exp spinal cord/ | **788757** |
| 4 | 2 or 3 | **1241661** |
| 5 | 1 and 4 | **8444** |
| 6 | limit 5 to dc=20240108-20250414 | **753** |

**Total Search Results 1002**

**With Duplicates Removed 878**

**Table 3: Reasons for full text exclusion**

| **Author (year)** | **Reason for exclusion** |
| --- | --- |
| Aanonsen et al. (1986) | No spinal cord injury animal model used – wrong population |
| Abdanipour et al. (2015) | No neurobehavioral outcome |
| Abematsu et al. (2012) | Conference abstract |
| Abramets et al. (1986) | Non-English language (Russian) |
| Alqinyah et al. (2017) | No spinal cord injury animal model used – wrong population |
| Araki et al. (2004) | No spinal cord injury animal model used – wrong population |
| Ault et al. (1978) | No spinal cord injury animal model used – wrong population |
| Bang et al. (2013) | No neurobehavioral outcome |
| Bao et al. (2008) | Wrong intervention - no HDAC inhibitor |
| Becker et al. (2019) | Conference abstract |
| Belmaker et al. (2004) | No spinal cord injury animal model used – wrong population |
| Cai et al. (2006) | No spinal cord injury animal model used – wrong population |
| Cartlidge et al. (1974) | No spinal cord injury animal model used – wrong population |
| Chen et al. (2014) | Wrong intervention |
| Chen et al. (2016) | Wrong study design – perspective article |
| Chen et al. (2019) | No neurobehavioral outcome |
| Chen et al. (2020) | No neurobehavioral outcome |
| Cheng et al. (2010) | Wrong study design – Editor’s choice |
| Cheng et al. (2022) | Wrong intervention - no HDAC inhibitor |
| Cho et al. (2013) | No spinal cord injury animal model used – wrong population |
| Cho et al. (2015) | No spinal cord injury animal model used – wrong population |
| Cobacho et al. (2009) | No spinal cord injury animal model used – wrong population |
| Curtis et al. (1958) | No spinal cord injury animal model used – wrong population |
| Denk et al. (2013) | No spinal cord injury animal model used – wrong population |
| Farkas et al. (2023) | Wrong intervention - no HDAC inhibitor |
| Feng et al. (2022) | Wrong intervention - no HDAC inhibitor |
| Finelli et al. (2013) | No neurobehavioral outcome |
| Fishman et al. (1997) | Abstract and full text unavailable |
| Fowell et al. (2007) | No spinal cord injury animal model used – wrong population |
| Fowell et al. (2008) | No spinal cord injury animal model used – wrong population |
| Grafova et al. (1994) | Full text unavailable |
| Hammond et al. (1992) | No spinal cord injury animal model used – wrong population |
| He et al. (2022) | Wrong intervention |
| He et al. (2022) | Wrong intervention |
| Hervera et al. (2019) | No neurobehavioral outcome |
| Jiang et al. (2023) | Wrong intervention |
| Jianwei et al. (2021) | Non-English language (Chinese) |
| Jing et al. (2021) | Wrong intervention |
| Kong et al. (2017) | No neurobehavioral outcome |
| Kong et al. (2020) | No neurobehavioral outcome |
| Lanza et al. (2018) | Conference/meeting abstract |
| Laumet et al. (2015) | No spinal cord injury animal model used – wrong population |
| Lee et al. (2014) | No neurobehavioral outcome |
| Li et al. (2014) | Full text unavailable |
| Li et al. (2023) | Wrong intervention - no HDAC inhibitor |
| Lin et al. (2019) | No neurobehavioral outcome |
| Liu et al. (2013) | No spinal cord injury animal model used – wrong population |
| Lopez et al. (2022) | Conference abstract |
| Lu et al. (2013) | Non-English language (Chinese) |
| Lu et al. (2019) | Wrong intervention - no HDAC inhibitor |
| Lu et al. (2023) | Wrong intervention |
| Maky et al. (1987) | Non-English language (Russian) |
| Mardi et al. (2017) | Full text unavailable |
| Marsh et al. (2002) | Abstract and full text unavailable |
| Naftchi et al. (1991) | Wrong intervention - no HDAC inhibitor |
| Nakashima et al. (2011) | Conference abstract |
| Nakashima et al. (2015) | Conference abstract |
| Nan et al. (2009) | No neurobehavioral outcome |
| Nita et al. (2013) | No spinal cord injury animal model used – wrong population |
| Niu et al. (2022) | Wrong intervention |
| Oruc et al. (2023) | No neurobehavioral outcome |
| Plunet et al. (2018) | Conference abstract |
| Rao et al. (2023) | Wrong intervention - no HDAC inhibitor |
| Rivieccio et al. (2009) | No spinal cord injury animal model used – wrong population |
| Romeo- Guitart et al. (2019) | No spinal cord injury animal model used – wrong population |
| Russo et al. (2016) | No spinal cord injury animal model used – wrong population |
| Su et al. (2014) | No neurobehavioral outcome |
| Su et al. (2016) | Wrong intervention - no HDAC inhibitor |
| Tapia et al. (2017) | Conference abstract |
| Throm et al. (2003) | Non-English language (German) |
| Throm et al. (2008) | Non-English language (German) |
| Tian et al. (2022) | Wrong intervention - no HDAC inhibitor |
| Tiraihi et al. (2013) | Conference abstract |
| Wang et al (2023) | Wrong intervention |
| Wang et al. (2016) | Conference abstract |
| Wang et al. (2017) | no neurobehavioral outcome |
| Wang et al. (2018) | Wrong intervention - no HDAC inhibitor |
| Wang et al. (2018) | Wrong intervention - no HDAC inhibitor |
| Wang et al. (2019) | Wrong intervention - no HDAC inhibitor |
| Wang et al. (2021) | Wrong intervention |
| Wei et al. (2023) | No spinal cord injury animal model used – wrong population |
| Xiao et al. (2022) | Wrong intervention - no HDAC inhibitor |
| Xu et al. (2023) | Wrong intervention - no HDAC inhibitor |
| Yu et al. (2019) | Wrong intervention - no HDAC inhibitor |
| Yun et al. (2020) | No neurobehavioral outcome |
| Zhang et al. (2019) | Conference abstract |
| Zhang et al. (2022) | No spinal cord injury animal model used – wrong population |
| Zhao et al. (2017) | Wrong intervention - no HDAC inhibitor |
| Zhao et al. (2021) | Wrong intervention - no HDAC inhibitor |
| Zhong et al. (2021) | Wrong intervention - no HDAC inhibitor |
| Zhong et al. (2023 | Wrong intervention |
| Zhou et al. (2017) | No neurobehavioral outcome |
| Zhou et al. (2020) | Wrong intervention |
| Zhu et al. (2022) | No spinal cord injury animal model used – wrong population |
| Zhang et al. (2024) | Wrong intervention - no HDAC inhibitor |
| Zylberberg et al. (2024) | Wrong intervention - no HDAC inhibitor |
| Oruc et al. (2024) | Full text unavailable |

**Table 4: Data extraction table.**

| **Author (year),**  **Location** | **Study characteristics** | **Sample characteristics** | **Injury model** | **Intervention** | **Outcomes assessed** | **Time of assessment** | **Statistical analysis used** | **Key findings** |
| --- | --- | --- | --- | --- | --- | --- | --- | --- |
| Abdanipour et al (2012)  Iran, Germany | 5 experimental groups | n=30  Sprague-Dawley rats  **Age:** not specified  **Sex:** female  **Weight:** 250-350g  **Comorbidities:** N/A | Traumatic SCI,  Contusion model using a 10g metal rod with 2mm diameter dropped from a height of 25mm  **Level:** T12-L1 | - Sham operated by laminectomy (n=6) - Untreated SCI (n=6) - SCI + 100 mg/kg VPA i.p. (n=6) - SCI + 200 mg/kg VPA i.p. (n=6) - SCI + 400 mg/kg VPA i.p. (n=6)   All VPA administered 3h after SCI, daily, for 7 days. | BBB locomotor scale | Post-SCI on days 3, 7, 14, 21, 28 | One-way ANOVA and Tukey’s post hoc analysis | - From day 14 onwards, there was a significant improvement among 200 and 400 mg/kg VPA-treated groups and other groups (sham, untreated SCI and 100mg/kg VPA; p<0.05) |
| Abematsu et al. (2010)  Japan | 7 experimental groups | n=46*  ICR mice  **Age:** 15 weeks  **Sex:** male  **Weight:** 37-45g  **Comorbidities:** N/A  *excludes mice which had incomplete injury – BBB locomotor score >0, 1 week after SCI: 2.4% of initial number of mice | Traumatic SCI,  Contusion model using 90kdyn SCI device  **Level:** T9 complete laminectomy, T10 partial laminectomy | - Saline-treated SCI (n=8) - VPA-treated SCI 150 mg/kg i.p. daily (n=8) - Neural stem cell transplant (GFP.LUC-NSC) + saline i.p. daily treated SCI (n=3) - Neural stem cell transplant (TR6.GFP.LUC-NSC) + saline i.p. daily treated SCI (n=6) - Neural stem cell transplant (GFP-NSC) + 150 mg/kg i.p. VPA daily (n=6) - Neural stem cell transplant (GFP.LUC-NSC) + 150 mg/kg i.p. VPA daily (n=9) - Neural stem cell transplant (TR6.GFP.LUC-NSC) + 150 mg/kg i.p. VPA daily (n=6)   Mice transplanted with NSC 1 week after SCI, VPA or saline given after transplant for 7 days.  NSCs were prepared from embryonic forebrains of 3 different Tg mouse lines ubiquitously expressing either GFP (GFP-Tg) (33), GFP and LUC (GFP.LUC-Tg), or GFP, LUC, and the diphtheria toxin (DT) receptor human heparin-binding EGF-like growth factor (TR6) (TR6.GFP.LUC-Tg) | BBB locomotor scale | Post-SCI starting 1 week after SCI and continuing for up to 14 weeks, assessed weekly | Repeated measures ANOVA | - From week 2 onwards, mice transplanted with neural stem cells that received VPA significantly improved their locomotor function compared to untreated SCI mice (p<0.001) - mice treated with VPA alone showed no further improvement compared with untreated mice |
| Chen et al. (2018)  China | 4 experimental groups, each had 4 subgroups (n=12) for 1-, 3-, 7- and 14-day time points, 6 rats in each group were euthanized for biological molecular and biochemical experiments and the remaining 6 were utilised for neurological and histological studies | n= 24  Wistar rats  **Age**: adult  **Sex**: male  **Weight:** 230-260g  **Comorbidities:** N/A | Traumatic SCI,  Contusion model using a 10g metal rod dropped from a height of 25mm  **Level:** T10 | - Sham operated by laminectomy at T9-10 level +DMSO (n=48) - Sham + VPA (n=48) - SCI+ VPA (n=48) - SCI+ DMSO (n=48)   VPA was administered app. 30min after SCI, at a dose 300 mg/kg/day i.p. diluted in DMSO, for 3 consecutive days.  DMSO was given in the same way as VPA. | - BBB locomotor scale - Inclined plane test | **BBB locomotor scale and inclined plane test:**  assessed on 1, 3, 7, 14 days post-SCI | **BBB score and inclined plane test:** one-way ANOVA with post hoc multiple comparisons using Student Newman-Keuls tests | - **BBB score:** the most significant improvement was seen 7 days post-SCI in VPA-treated group (p<0.05) - **Inclined plane test:** maximum angles were higher in VPA-treated group than in the untreated group (27.44 ± 2.48 vs 19.75 ± 1.62, p < 0.05) |
| Chen et al. (2023)  China | 4 experimental groups | n=24  Sprague-Dawley rats  **Age:** adult, not further specified  **Sex:** male  **Weight:** 220-250g  **Comorbidities:** N/A | Traumatic SCI,  Contusion model using a 10g metal rod dropped from a height of 25mm  **Level:** T10 | - Sham operated by laminectomy (n=6) - Untreated SCI (n=6) - SCI+vehicle (n=6) - SCI+ RGFP966 10 mg/kg/day i.p. 30 min after SCI for 3 consecutive days (n=6)   Vehicle was administered in the same way as RGFP966 | BBB locomotor scale | Post-SCI on days 1, 3, 7, 14 for experiment 1 | Repeated-measures ANOVA | - BBB scores of rats in the 3 SCI groups gradually increased over time with a significant improvement in the RGFP966-treated group 7 days post-SCI (p<0.01 vs SCI+ vehicle group) |
| Dai et al. (2021)  China | 5 experimental groups | n=40  C57BL/6 mice  **Age:** 8 weeks  **Sex:** male  **Weight**: 23-27g  **Comorbidities:** N/A | Traumatic SCI,  epidural compression of the spinal cord with a 24-g closure force applied for 1 min  **Level:** T6-T7 | - Sham operated by laminectomy + vehicle (n=8) - SCI + vehicle (n=8) - SCI + Entinostat 1mg/kg, gavage (n=8) - SCI + Entinostat 5mg/kg, gavage (n=8) - SCI + Entinostat 10mg/kg, gavage (n=8)   Entinostat or equal volumes of PBS (vehicle) were administered 4h and then 24h and 48h after SCI. | - Forelimb grip strength - Basso Mouse Scale | **Forelimb grip strength and**  **BMS:** assessed pre-SCI and post-SCI on days 1, 2, 3, 7, 14, 21, 28 | - **Forelimb grip strength:** two-way ANOVA followed by Tukey’s multiple comparisons test - **BMS**: one-way ANOVA followed by Tukey’s multiple comparisons test | - **BMS score**: 7-28 days post-SCI: BMS score was significantly improved in the Entinostat 5mg/kg and 10mg/kg groups compared to vehicle-treated SCI group (p<0.05) - **Forelimb grip strength:** on days 7-28 post-SCI: Entinostat improved forelimb grip strength in 5mg/kg and 10mg/kg groups (p<0.05) |
| Darvishi et al. (2014)  Iran | 22 experimental groups | n= 200  Sprague-Dawley rats  **Age:** not specified  **Sex:** female  **Weight**: 220-240g  **Comorbidities:** N/A | Traumatic SCI,  Contusion model using the NYU weight drop device with a 10g metal rod 2.5 mm diameter dropped from a height of 12.5mm  **Level**: T8 | - Sham operated by laminectomy - negative control: SCI+ saline (SC injected with normal saline) - SCI + VPA i.p. at 2h post-SCI - SCI + VPA i.p. at 6h post-SCI - SCI + VPA i.p. at 12h post-SCI - SCI + VPA i.p. at 24h post-SCI   Each of the SCI + VPA groups was subdivided into 5 different VPA doses: 150, 200, 300, 400, 500 mg/kg (n=10 in each subgroup) | BBB locomotor scale | assessed daily 10 days pre-SCI and post-SCI on days 1, 3, 7 and then twice a week for 2 weeks and afterwards once a week for 12 weeks | Repeated measures ANOVA with Tukey’s test post hoc analysis | - the animals treated with 500 mg/kg at all delivery times died - the optimal dose was 300 mg/kg at 12h post-SCI where the BBB score was significantly higher compared to all other experimental groups at all doses used in the therapy protocol (p<0.05) and to untreated controls (p<0.05) |
| Hao et al. (2013)  China | 3 experimental groups | n= 15  Sprague-Dawley rats  **Age**: adult  **Sex:** female  **Weight**: 220-250g  **Comorbidities:** N/A | Traumatic SCI,  Contusion model using modified New York University impactor with a 10g metal rod 2.5 mm diameter dropped from a height of 25 mm  **Level:** T10 | - Sham operated by laminectomy (n=5) - SCI + saline i.p. twice daily starting immediately after SCI and continuing for 2 weeks (n=5) - SCI + VPA 300mg/kg i.p. twice daily starting immediately after SCI and continuing for 2 weeks (n=5) | BBB locomotor scale | Assessed once a week for 6 weeks post-SCI | Repeated measures ANOVA followed by the Tukey-Kramer test | - in weeks 4-6 post-SCI, BBB scores in the VPA-treated animals were consistently and significantly higher than those in the vehicle-treated rats (p<0.05) |
| He et al. (2017)  China | 4 experimental groups | n=32  Sprague-Dawley rats  **Age:** adult  **Sex**: female  **Weight:** 220-250g  **Comorbidities:** N/A | Traumatic SCI,  Compression of spinal cord with a vascular clip (15 g) for 2 minutes  **Level:** T9 | - Sham operated by laminectomy (rats were from the control group of the diabetes model) (n=8) - SCI group (rats were from the control group of the diabetes model) (n=8) - DM + SCI + vehicle (DMSO) at equal doses to the 4-PBA group starting immediately after SCI and repeating daily for 4 weeks (n=8) - DM + SCI +100 mg/kg i.p. 4-PBA starting immediately after SCI and repeating daily for 4 weeks (n=8)   Rats in the SCI, DM + SCI and DM + SCI + 4-PBA groups were subjected to SCI 4 weeks after streptozotocin injection. | - BBB locomotor scale - Inclined plane test | **BBB and inclined plane test:** assessed post-SCI at days 1, 3, 7, 14, 28 | BBB and inclined plane test: two-way ANOVA followed by Bonferroni post-hoc comparison test | - **BBB locomotor score:** 4-PBA significantly improved locomotor function in diabetic rats 28 days post- SCI (p<0.05 vs diabetic untreated SCI rats) - **Inclined plane test:** 4-PBA significantly improved locomotor function in diabetic rats after SCI (p<0.05 vs diabetic untreated |
| Hendrix et al. (2020)  Belgium | 3 experimental groups | n= 21-27*  Balb/c mice  **Age:** 10-week-old  **Sex:** female  **Weight:** not specified  **Comorbidities:** N/A  *unclear | Traumatic SCI  T-cut spinal cord hemisection injury using iridectomy scissors  **Level:** T8 | - SCI + VPA 250 mg/kg i.p. starting 6h from the SCI for 5 consecutive days (n=7-9) - SCI + PCI-34051 20 mg/kg i.p. starting 6h from the SCI for 5 consecutive days (n=7-9) - SCI + vehicle containing 9 % vehicle solution (30 % PEG400, 5% propylene glycol 0.5% tween-80 in NaCl) i.p. starting 6h from the SCI for 5 consecutive days (n=7-9)   Frequency of administration was not specified. | Basso Mouse Scale | Assessed starting 1 day after SCI until 35 days after SCI, for the first 8 days mice were scored daily, afterwards every second day | two-way ANOVA for repeated measurements with Bonferroni’s post hoc test for multiple comparisons | - no effect of VPA or PCI-34051 on functional recovery |
| Jafarimanesh et al. (2023)  Iran | 5 experimental groups | n=35  Sprague-Dawley rats  **Age:** not specified  **Sex:** not specified  **Weight:** 250g (mean body weight)  **Comorbidities:** N/A | Traumatic SCI  hemisection spinal cord injury  **Level:** T9-10 | - SCI + Alg-Cs/Npch (received Alg/Cs containing Cs nanoparticles) (n=7) - SCI + Alg-Cs/Npch/hEnSCs (received Alg-Cs containing Cs nanoparticles seeded with hEnSCs) (n=7) - SCI + Alg-Cs/ Npch/Val (received Alg-Cs containing Cs nanoparticles loaded with Val) (n=7) - SCI + Alg-Cs/Npch/Val/hEnScs (received Alg-Cs containing Cs nanoparticles loaded with Val and hEnSCs) (n=7) - Untreated SCI (n=7)   For all treated groups, hydrogels were implanted into the lesion site after SCI.  Hydrogel components:   - Valproic acid (Val) encapsulated within hybrid of alginate (Alg)-chitosan (Cs) (Alg-Cs) hydrogel containing Cs nanoparticle (Npch) with/without human endometrial stem cells (hEnSC). | BBB locomotor scale | Assessed pre-SCI and post-SCI on days 1, 3, 5, 7, 14, 28 | Unclear | - The highest BBB score was detected in hydrogel with human endometrial stem cells + valproate group (7.1 ± 4.5) followed by hydrogel with valproate (6.4 ± 5.1), hydrogel with human endometrial stem cells (6.1 ± 4.8), hydrogel (5.714.4), and control (2.1 ± 2.90) groups*   *unclear statistical significance |
| Kuboyama et al. (2017)  USA | 2 experimental groups | n=16  C57BL/6J mice  **Age:** 5-7 weeks  **Sex:** female  **Weight:** not specified  **Comorbidities:** N/A | Traumatic SCI  Contusion injury using an infinite horizon impactor 70 kDyn force (neurobehavioral outcomes were assessed only in this one)    **Level:** T8 | - SCI + vehicle administered at 2, 24 and 48h after SCI (n=10) - SCI + RGFP966 10 mg/kg i.p. administered at 2, 24 and 48h after SCI (n=6) | - Basso mouse scale score - Toyama mouse score (TMS) | **BMS and TMS:** measured every day after SCI for 30 days | **BMS and TMS:** repeated measures, two-way ANOVA with Bonferroni post hoc correction. | - **Both BMS and TMS:** RGFP966-treated animals exhibited improved BMS and TMS scores compared to the vehicle-treated cohort (p < 0.001) |
| Lanza et al. (2019)  Italy | 5 experimental groups | n=50  CD1 mice  **Age:** 6-8 weeks  **Sex:** male  **Weight:** 25-30g  **Comorbidities:** N/A | Traumatic SCI,  Extradural compression with aneurysm clip (24 g) for 1 minute.  **Level:** T6-7 | - Sham + vehicle (oral saline) group (n=10) - SCI + vehicle (saline; n=10) - SCI + sodium butyrate 10 mg/kg oral 1h and 6h after SCI (n=10) - SCI + sodium butyrate 30 mg/kg oral, 1h and 6h after SCI (n=10) - SCI + sodium butyrate 100 mg/kg oral, 1h and 6h after SCI (n=10) | Basso Mouse Scale | Assessed daily until day 9 | Mann–Whitney test | - 30 and 100 mg/kg dose groups demonstrated significant improvement in BMS scores (for 100 mg/kg dose p<0.001 and for 30 mg/kg p<0.05 when compared to SCI group) |
| Lee et al. (2012)  Korea | 2 experimental groups | n= 30  Sprague-Dawley rats  **Age:** adult  **Sex:** male  **Weight**: 250-300g  **Comorbidities:** N/A | Traumatic SCI  Contusion model either moderate or moderately severe contusion using a 25 gm-cm or 50 gm-cm impactor device respectively  **Level:** T9-10 | - SCI + vehicle (PBS) equal volume as VPA injection subcutaneously at the same times as VPA (n=15) - SCI + 300 mg/kg VPA immediately after SCI and then the same dose injected subcutaneously every 12h for 5 days (n= 15) | - BBB locomotor scale - Horizontal grid walk test - Footprint analysis | - **BBB:** evaluated for 5 weeks after SCI, weekly - **Both horizontal grid walk test and footprint analysis:** at 35 days post-SCI | - **BBB:** Repeated measures ANOVA (time vs. treatment) with Tukey’s multiple comparison as post hoc analysis - **Horizontal grid walk test:** Student’s t-test - **Footprint analysis**: no statistical analysis | - **BBB score:** at 35 days in the VPA group the BBB score was significantly higher compared to the vehicle-treated group (p<0.01) - **Horizontal grid walk test:** The number of foot falls on the grid walk in VPA-treated groups was significantly lower compared to the vehicle-treated group (p<0.05) - **Footprint analysis:** VPA-treated rats showed fairly consistent forelimb-hindlimb coordination and very little toe dragging compared to vehicle-treated animals that showed inconsistent coordination and extensive drags |
| Lu et al. (2013)  Taiwan | 2 experimental groups | n=14  Sprague-Dawley rats  **Age:** adult  **Sex:** female  **Weight:** 220-280g  **Comorbidities:** N/A | Traumatic SCI  Contusion model  using a 10g rod dropped from a height of 50mm while keeping the dura intact  **Level**: T9/10 | - SCI + vehicle (PBS) delivered via an Alzet osmotic pump prefilled with PBS with a delivery rate of 1.0 μl/hr and a 3-day duration was placed 5-10 min after SCI slightly proximal to the LC and about 0.7–0.8 mm below the dura (n= 6) - SCI + VPA delivered via an Alzet osmotic pump prefilled with 1.5 μg VPA with a delivery rate of 1.0 μl/hr and a 3-day duration was placed 5-10 min after SCI slightly proximal to the LC and about 0.7–0.8 mm below the dura (n= 8) | BBB locomotor scale | on days 3, 5, 7, 9, 12, 14, 16, 18, 21, 31 post-SCI | Two-way ANOVA | - By day 31 post-SCI BBB score in VPA-treated rats showed continuous improvement compared to vehicle-treated SCI rats (p<0.05) |
| Luo et al. (2023)  China | 5 experimental groups | n=25  Sprague-Dawley rats  **Age:** adult  **Sex:** male  **Weight:** 250-300g  **Comorbidities:** N/A | Ischemia/Reperfusion Injury (I/R)  Aortic clamping between the left and right renal arteries with a non-invasive artery clamp for 85 minutes.  **Level:** N/A | - Sham group without ischemia, the abdomen was opened to expose the abdominal aorta, but without clamping + vehicle i.p. for 5 days before R/I (n=5) (n=10) - I/R group + vehicle i.p. for 5 days before R/I (n=5) - I/ R+Xe* + vehicle i.p. for 5 days before R/I (n=5) - I/R+ 50 mg/kg/day 4-PBA i.p. for 5 days before R/I (n=5) - I/R + Xe + 50 mg/kg/day 4-PBA i.p. for 5 days before R/I* (n=5)   *(50% nitrogen and 50% Xe in the premixed gas), oxygen and premixed gas were blended and supplied to the animals through the small animal ventilator. After 1 h of reperfusion, the rats inhaled 50 vol% Xe and 50 vol% oxygen for 1 h | - BBB locomotor scale - Tarlov scoring system | **Both BBB score and Tarlov score:** assessed over 3 min postreperfusion for 4h | **Both BBB score and Tarlov score:** One-way ANOVA followed by the Least Significance Difference post hoc test | - **Both BBB score and Tarlov score:** hind limb locomotor function after SCIRI were markedly ameliorated by 4-PBA treatment (p < 0.05) |
| Lv et al. (2012)  USA, China | 3 experimental groups | n= 36  Wistar rats  **Age:** not specified  **Sex**: female  **Weight:** 200-230g  **Comorbidities:** N/A | Traumatic SCI  Contusion model  using an NYU impactor device to drop a 10g rod from a height of 12.5mm  **Level:** T9 | - Sham operated by laminectomy (n= 12) - SCI + saline i.p. twice a day starting 8h after surgery and continuing for 1 week, volume of saline was the same as for VPA (n=12) - SCI + VPA 300 mg/kg (dissolved in saline) i.p. twice a day starting 8h after surgery and continuing for 1 week (n= 12) | - BBB locomotor scale - Footprint analysis | **Both BBB score and footprint analysis**: for 6 weeks post-SCI, weekly | - **BBB score:** repeated measures ANOVA followed by the Tukey–Kramer test - **Foorprint analysis:** one-way ANOVA followed by the Bonferroni post hoc test was used | - **BBB score**: by week 4, the animals receiving VPA scored significantly higher compared to untreated animals (p<0.05) - **Footprint analysis:** compared to controls, rats from VPA group showed smaller rotation angles and decreased base of support (p<0.05) |
| Lv et al. (2011)  China | 3 experimental groups | n= 36  Wistar rats  **Age:** not specified  **Sex**: female  **Weight**: 200-220g  **Comorbidities:** N/A | Traumatic SCI  Contusion model  using an NYU impactor device to drop a rod from a height of 12.5mm (weight of the rod not specified)  **Level:** T8 | - Sham operated by laminectomy (n=12) - SCI + vehicle i.p. twice a day staring immediately after SCI and continuing for 2 weeks (n=12) - SCI + VPA 300 mg/kg, dissolved in saline, i.p. twice a day staring immediately after SCI and continuing for 2 weeks (n=12) | - BBB locomotor scale - Narrow beam test - Footprint analysis | - **BBB score:** after baseline testing, the animals were tested once a week for 8 weeks post-SCI - **Both narrow beam test and footprint analysis:** 8 weeks post-SCI | - **BBB score:** Repeated measures ANOVA followed by the Tukey–Kramer test - **Narrow beam test and footprint analysis:** independent samples t-test | - BBB score: VPA-treated animals showed significant differences from controls in BBB score starting between 4 weeks and until 8 (p<0.01) - Narrow beam test: significantly higher in VPA-treated animals compared to controls (p=0.037) - Footprint analysis: significantly reduced foot exorotation and base of support in VPA-treated animals compared to controls (p<0.01) |
| Mardi et al. (2021)  Iran | 4 experimental groups | n= 32  Wistar rats  **Age:** 18-12 weeks  **Sex:** female  **Weight:** 200-250g  **Comorbidities:** N/A | Traumatic SCI  Severe contusion model  using a weight drop device  **Level:** not specified | - Sham operated by laminectomy (n=8) - Untreated SCI (n=8) - SCI + saline 0.5 ml i.p. administered starting 3h after SCI and continued once daily for 7 days (n=8) - SCI + VPA 400 mg/kg i.p. administered starting 3h after SCI and continued once daily for 7 days (n=8) | BBB locomotor scale | assessed post-SCI at days 3, 7, 14, 21, 28 | Not reported | - significant increase at 21- and 28-days post SCI in the VPA-treated group compared to the untreated SCI group |
| Mizukami et al. (2010)  Japan | 3 experimental groups | n= 18  Japanese white rabbits  **Age:** not specified  **Sex:** female  **Weight:** 2.5-3.0 kg  **Comorbidities:** N/A | Ischemia/Reperfusion Injury (I/R),  the abdominal aorta just distal to the left renal artery and just proximal to the iliac bifurcation was cross-clamped and isolated for 15min to produce spinal cord ischaemia  **Level:** N/A | - sham operation control group with only aortic exposure but without transient ischaemia (n=6) - transient ischaemia + vehicle (saline) 1ml/kg IV from 30 min before induction of ischaemia until 30min after reperfusion (n=6) - transient ischaemia + 4-PBA 4mg/ml in saline vehicle, 15 mg/kg/h IV from 30 min before induction of ischaemia until 30min after reperfusion (n=6)   Total operation time ranged from 1.5-2.5h.  Total amount of 4-PBA administration ranged from 46.9 mg to 56.3 mg. | Tarlov score | assessed at 8 hours, and 2 and 7 days after reperfusion | One-way analysis of variance*  *unclear | - Tarlov score was significantly higher in 4-PBA treated group than vehicle-treated SCI group on days 2 and 7 post I/R SCI (p<0.0001) - There was no significant difference between the treated group and sham control at all assessed timepoints |
| Penas et al. (2011)  Spain | 2 experimental groups | n= 12  Sprague Dawley rats  **Age:** 11 weeks  **Sex:** female  **Weight:** 240-270g  **Comorbidities:** N/A | Traumatic SCI  Contusion model  using the Infinite Horizon impactor device at 150 kDyn or 250 kDyn  **Level:** T8 | - SCI 250 kDyn + vehicle saline i.p. 3h after surgery and then every 24h for the first week post-surgery and then every 24h for the next 2 weeks (n=6) - SCI 250 kDyn + VPA administered as an initial bolus of 150 mg/kg, i.p. 3 h after surgery, followed by 300 mg/kg every 12 h during the first week post-surgery, and then every 24 h for the next 2 weeks. (n=6) | BBB locomotor scale | assessed pre- SCI and post-SCI at days 3, 7, 14, 28 | Two-way ANOVA followed by Bonferroni post-hoc tests | - At 35 days post-SCI, the VPA-treated animals showed significantly higher BBB scores compared to the saline-treated controls (p<0.0012)*   *unclear |
| Qi et al. (2018)  China | 2 experimental groups | n= 30  C57BL/6J mice  **Age:** 8 weeks  **Sex**: male  **Weight**: not specified  **Comorbidities:** N/A | Traumatic SCI  Contusion model  using the Infinite Horizon impactor device at 60 kDyn  **Level:** T9/10 | - SCI + vehicle (DMSO) i.p. daily starting immediately after SCI (n=15) - SCI + TMP269 50 mg/kg i.p. daily starting immediately after SCI (n=15) | BBB locomotor scale | Assessed twice daily for the first week post-SCI and weekly afterwards until 6 weeks post-SCI | Unpaired two-tailed Student's t-test | - On day 42 post-SCI the BBB score of TMP269-treated group were lower than those of control group |
| Qin et al. (2024)  China | 3 experimental groups | n=15  mice, species not specified  **Age:** not specified  **Sex**: not specified  **Weight:** not specified  **Comorbidities:** N/A | Traumatic SCI  Contusion model  using modified Allen’s weight drop apparatus, dropping a 10g weight at a vertical height of 25mm  **Level**: T10 | - SCI + NC-exosomes (exosomes derived from negative control of miR-34a-5p treated EGFR+NSCs) solid hydrogel patches containing exosomes (10 mg/mL) placed upon the injury site (n=5) - SCI + miR-34a-5pIN-Exos (exosomes derived from miR-34a-5p-knockdown EGFR+NSCs) solid hydrogel patches containing exosomes (10 mg/mL) placed upon the injury site (n=5) - SCI + miR-34a-5pIN-Exos + SW-100 solid hydrogel patches containing exosomes (10 mg/mL) placed upon the injury site (n=5) | Basso Mouse Scale | before SCI, immediately after, and on days 1, 3, 7, 14, 21, 28 post-SCI | Repeated-measures two-way ANOVA | - the presence of SW-100 partly rescued the functional effects of miR-34a-5pIN-Exos and improved the BMS score compared to the miR-34a-5pIN -Exos treatment alone (day 14 post-SCI: p<0.05, day 21, 28 post-SCI: p<0.01) |
| Reis et al. (2020)  Brasil | 3 experimental groups | n= 18  Wistar rats  **Age:** 2 months  **Sex**: male  **Weight:** 250-300g  **Comorbidities:** N/A | Traumatic SCI  Right-side hemisection by placing a 28-gauge needle dorsi-ventrally at the midline of the cord and pulling it laterally to ensure a complete hemisection  **Level:** T10 | - SCI no scaffold (n=6) - SCI + PLGA scaffold with diameter of 2 mm and a thickness of approximately 300 mm, was carefully placed into the hemisected gap immediately after the SCI (n=6) - SCI + VPA/PLGA scaffold with diameter of 2 mm and a thickness of approximately 300 mm, was carefully placed into the hemisected gap immediately after the SCI (n=6)   The core of the fibres contained 25 mg/mL VPA sodium salt | BBB locomotor scale | Assessed 2 days post-SCI and then weekly until 6^th^ week post-SCI | One-way ANOVA, followed by the Bonferroni post-hoc test | - there was no significant difference at any of the weekly time points between the control and VPA-treated group |
| Sanchez et al. (2018)  Belgium, Ireland | 3 experimental groups | n= 54-69*  Balb/c mice  **Age:** 10 weeks  **Sex**: female  **Weight:** not specified  **Comorbidities:** N/A  *unclear | Traumatic SCI,  T-cut spinal cord hemisection injury using iridectomy scissors to transect left and right dorsal funiculus, the dorsal horns and the ventral funiculus.  **Level:** T8 | - SCI + RGFP966 (10 mg/kg, dissolved in 7.7% DMSO in NaCl) i.p. starting 2h after SCI and continuing for 3 consecutive days (n ∼ 18-23) - SCI + scriptaid (3.5 mg/kg, dissolved in 0.9% DMSO in NaCl) i.p. starting 2h after SCI and continuing for 3 consecutive days (n ∼ 18-23) - SCI + vehicle containing 0.9% DMSO in NaCl i.p. starting 2h after SCI and continuing for 3 consecutive days (n ∼ 18-23) | Basso Mouse Scale | from 1 day post-SCI, in the first week scored daily and then every other day until day 27 post-SCI | Two-way ANOVA for repeated measurements with Bonferroni’s post hoc test for multiple comparisons | - no significant differences in BMS scores between the mice treated with **scriptaid** and the vehicle control group - no significant differences in BMS scores between mice treated with **RGFP966** and vehicle-treated controls |
| Seira et al. (2020)  Canada, China, USA | 4 experimental groups | n= 28  C57Bl6 Pten floxed mice  **Age:** 4 weeks for the young mice group and 7-8 months for old mice group  **Sex:** not specified  **Weight:** not specified  **Comorbidities:** N/A | Traumatic SCI,  Dorsolateral funiculus (DLF) crush with  fine forceps modified for this purpose by grinding their blades to a width of ~200 mm, one of the prongs was inserted into the dorsal horn gray matter (~1 mm deep) while the other prong was on the lateral surface of the spinal cord. The forceps were closed and held for 15 seconds to crush the dorsolateral funiculus containing the RST (and this was repeated once).  **Level:** C4-C5 | Analysis of mice which AAV transduction of GFP-CRE or GFP failed (the procedure for transduction was the same as above):  4-week-old mice – young group:   - AAV + SCI + TSA 7mg/kg i.p. daily starting 2h post-SCI and continuing for 7 consecutive days (n=5) - AAV + SCI + vehicle (1:10 DMSO/saline) i.p. daily starting 2h post-SCI and continuing for 7 consecutive days (n=6)   7-8 months old mice – old group:   - AAV + SCI + TSA 7mg/kg i.p. daily starting 2h post-SCI and continuing for 7 consecutive days (n=8) - AAV + SCI + vehicle (1:10 DMSO/saline) i.p. daily starting 2h post-SCI and continuing for 7 consecutive days (n=9) | - Cylinder test - Horizontal ladder test | - **Cylinder test:** assessed post-SCI on days 3, 8, 15, 28 and 8 and 12 weeks post-SCI - **Horizontal ladder test:** assessed pre-SCI to obtain preinjury scores and then post-SCI on days 3, 8, 15, 28 and 8 and 12 weeks post-SCI | - **Cylinder test:** two-way ANOVA with Fisher LSD post-hoc test - **Horizontal ladder test:** unpaired t-test with post-hoc HolmeSidak method (when indicated) and two-way ANOVA with Fisher LSD post-hoc test (when indicated) | - **Cylinder test**: in old animals, significant differences were observed between the aged TSA and VH groups at 8DPI for left/ ipsilateral paw, 28 DPI for the right/contralateral paw (p=0.02) and 28DPI for both paws; significant differences were seen between the treatment groups for the percentage of ipsilateral paw placements (15days post-SCI: p < 0.05 and 28 days post-SCI p < 0.001) and in percentage of contralateral paw placements (15, 28 days post-SCI, 12 weeks post-SCI, all p < 0.05); in young animals no significant differences were found between treatment and control groups - **Horizontal ladder test:** While TSA treatment seemed to induce a higher number of errors in older mice, the opposite was observed in young mice (p < 0.01 for old mice; p < 0.05 for young mice); TSA treatment had no effect on the CE at any other time point after injury, although we observed a sustained trend toward an increase (old) and a decrease (young) in errors until 28 DPI in both groups; No deficits were found for the right/contralateral forelimb after treatments |
| Ulas et al. (2023)  Turkey | 2 experimental groups | n= 16  Wistar rats  **Age:** not specified  **Sex:** male  **Weight:** 220-230g  **Comorbidities:** N/A | Ischaemia/reperfusion injury  Cross-compression of the aorta using an  atraumatic microvascular clamp placed along the infrarenal and iliac bifurcation parts of the abdominal aorta for 45 minutes.  **Level:** N/A | - SCI + 1ml saline i.p. single dose, time of administration not specified (n=8) - SCI + VPA 300 mg/kg i.p. single dose, time of administration not specified (n=8) | - BBB locomotor scale - Inclined plane test | **Both BBB score and inclined plane test:** at 1^st^, 6^th^, 12th, 24^th^, 48th hour post-SCI | **BBB and inclined plane test:** One-way ANOVA*  *unclear | - **BBB score:** compared with the rats in the SCI + saline group, VPA-treated rats showed higher BBB scores at each time point after 12h (p<0.05) - **Inclined plane test:** in the VPA-treated group, the angle of incline was increased, compared with the SCI + saline group at each time point (p<0.05) |
| Wang et al. (2020)  China | 5 experimental groups | n=52*  Sprague-Dawley rats  **Age:** adult  **Sex:** male  **Weight:** 180-220g  **Comorbidities:** N/A  *Rats that died for any reasons were excluded from the experiment, and a new one was added to the study. | Traumatic SCI  Contusion model established  using a weight drop apparatus, dropping a 5g rod at a vertical height of 80mm  **Level**: T10 | - Sham operated by laminectomy (n=9) - SCI + saline IV (n=10) - CN-treated SCI rats administered IV 15 mg/kg CN (chitosan nanoparticles) concentration of CN starting 1h post-SCI and continuing daily for 5 days (n=10) - SCI + valproic acid IV 80 mg/kg starting 1h post-SCI and continuing daily for 5 days (n=12) - SCI + VA-CN 15 mg/kg IV starting 1h post-SCI and continuing daily for 5 days (n=11)   IV injections were into the tail vein. | BBB locomotor scale  Rats with perineal infections, limb wounds, or tail and foot grazing were eliminated from the test (n=6) | days 1, 3, 7, 14, 28 post-SCI | ANOVA analysis of variance | - in the group treated with chitosan nanoparticles containing VPA for one week, the BBB scores were significantly increased compared with the SCI group (p<0.05) |
| Wang et al. (2021)  China | 5 experimental groups | n=45  Sprague-Dawley rats  **Age:** adult  **Sex:** male  **Weight:** 180-220g  **Comorbidities:** N/A | Traumatic SCI  Contusion model established  using a weight drop apparatus, dropping a 5g rod at a vertical height of 80mm    **Level:** T10 | - Sham operated by laminectomy (n=7) - SCI (n=8) - SCI + 15 mg/kg CN (chitosan nanoparticles) IV starting 1h after SCI and continued daily (n=10) - SCI + valproic acid 80 mg/kg IV starting 1h after SCI and continued daily (n=10) - SCI + CN-VA 15 mg/kg IV starting 1h after SCI and continued daily (n=10) | BBB locomotor scale | Assessed at 1, 2, 3 and 4 weeks after SCI | A one-way analysis of variance (ANOVA) followed by Dunnett’s test | - the BBB scores were significantly increased in the group treated with VPA containing chitosan nanoparticles when compared with the SCI control group (p<0.05 at 1 and 2 weeks post-SCI**,** p<0.01 at 3 and 4 weeks post-SCI) |
| Yu et al. (2012)  Korea | 3 experimental groups | n=36  Sprague-Dawley rats  **Age:** adult  **Sex:** male  **Weight:** 290-310g  **Comorbidities:** N/A | Traumatic SCI,  Spinal cord compression with a modified aneurysm clip with a closing force of 30 grams, the clip was rapidly released from the applicator and applied vertically onto the exposed spinal cord for 2min  **Level:** T9 | - Sham operated by laminectomy (n=12) - SCI + VPA 200 mg/kg i.p. twice daily at 12h intervals for 7 days (n=12) - SCI + saline 1ml i.p. twice daily at 12h intervals for 7 days (n=12) | BBB locomotor scale | Assessed each week for 2 weeks after the surgery | Repeated measures ANOVA | - There was a significant increase in BBB scores at day 7, 10 and 14 in the VPA-treated group compared to the saline-treated control (p<0.05) |
| Zhang et al. (2021)  China | 5 experimental groups | n=24  Sprague-Dawley rats  **Age:** adult  **Sex:** male  **Weight:** 180-220g  **Comorbidities:** N/A | Traumatic SCI  Contusion model established  using a modified Allen’s weight drop apparatus, dropping a 10g rod at a vertical height of 25mm  **Level:** T10 | - Sham operated by laminectomy (n=6) - SCI + 4-PBA (n=6) - SCI + NC-agomir intrathecal on day 0, 1, 2 after spinal cord surgery (n=6) - SCI + miR-211−5p-agomir 100 nM/mL, 50 μL intrathecal on day 0, 1, 2 after spinal cord surgery (n=6) - SCI + 4-PBA 200 µM, 10 μL intrathecal on day 0, 1, 2 after spinal cord surgery | BBB locomotor scale | Assessed on the 7^th^ day after SCI | One-way ANOVA followed by Tukey’s multiple comparison test | - 4-PBA significantly increased the BBB score in SCI rats compared to controls (p<0.05) |
| Zhang et al. (2018)  Japan | 2 experimental groups | n= 43  C57BL/6 J mice  **Age:** 8 weeks  **Sex:** not specified  **Weight**: not specified  **Comorbidities:** N/A | Traumatic SCI,  Dorsal hemi-section using a surgical blade at a depth of 1.0 mm. Avoiding the remaining portion of the lateral CST, the surgical blade was passed through the dorsal spinal cord several times to create a lesion that extended downward to the central canal.  **Level:** T8 | - SCI + 10mg/kg CI-994 i.p. at 3h after SCI and then once daily for 14 days (in BMS test n=22, in the remaining neurobehavioral tests n=16) - SCI + DMSO (no information provided on frequency, route of administration and start of administration) (in BMS test n=21, in the remaining neurobehavioral tests n=15) | - BMS score - narrow beam walk test - horizontal grid walk test - ladder walk test inclined plane test | - **BMS:**   assessed just before surgery and post-SCI at 1, 3, 7, 14, 21, 28, 35, 42   - **Narrow beam walk test:** assessed just before surgery and post-SCI on days 7, 14, 21, 28, 35, 42 - **Horizontal grid walk test:** assessed just before surgery and post-SCI on days 14, 21, 28, 35, 42 - **Ladder walk test:** tests started 2 weeks post-SCI and continued once a week for additional 4 weeks - **Inclined plane test:** assessed pre-SCI, and post-SCI on days 7, 14, 21, 28, 35, 42 | **BMS, narrow beam walk test, horizontal grid walk test, ladder walk test, inclined plane test:**  Two-way repeated measures ANOVA with Tukey-Kramer test | - **BMS:** 7 days post-SCI, CI-994-treated mice exhibited higher BMS scores (p<0.01), on days 14 and 21 p<0.05 and on days 28, 35 and 42 p<0.01 compared to vehicle-treated controls - **Narrow beam walk test:** there was significant improvement in CI-994 treated mice on days 14, 35 (p<0.05) and days 28 and 42 post-SCI (p<0.01) compared to vehicle-treated controls - **Horizontal grid walk test**: there was a significant reduction in percentage foot slips at day 14 post-SCI (p<0.05) compared to vehicle-treated controls - **Ladder walk test**: there was a significant reduction in percentage foot slips on days 14, 21, 28, 35, 42 post-SCI (p<0.01) compared to vehicle-treated controls - **Inclined plane test:** there was no significant difference between the treatment and control in the inclined plane test |
| Zheng et al. (2020)  China | 5 experimental groups | n=50  C57BL/6OlaCnc mice  **Age**: 8 weeks  **Sex:** male  **Weight**: 20-25g  **Comorbidities:** N/A | Traumatic SCI  Contusion model established  By dropping a 10g weight at a vertical height of 15mm  **Level:** T10 | - Sham operated by laminectomy (n=10) - SCI (n=10) - SCI + tubastatin A 50 mg/kg i.p. daily (n=10) - SCI + Baf-A1 0.3 mg/kg daily starting 3 days before surgery (n=10) - SCI + tubastatin A 50 mg/kg + Baf-A1 0.3 mg/kg both i.p. and injected daily (n=10)   Tubastatin A injections started after SCI and continued for 28 days although that is unclear.  Baf-A1 injections started 3 days before SCI and continued until 28 days post-SCI although that is unclear  15 mice died after SCI | - Basso Mouse Scale - Footprint analysis | **BMS and footprint analysis**: assessed post-SCI on days 0, 1, 3, 7, 14, 28 | - **BMS:** One way ANOVA with Tukey’s multiple comparisons test (for BMS score analysis at day 28 only); two-way ANOVA analysis followed by Tukey's multiple comparison test was performed for all time points - **Footprint analysis**: no statistical analysis | - **BMS:** significant improvement in BMS score in Tubastatin A-treated animals at 14 and days post-SCI (14 days: p<0.05; 28 days: p<0.001) compared to untreated-SCI group - **Footprint analysis:** there was improvement in the footprint patterns in Tubastatin A -treated animals compared to untreated SCI group |
| Zhou et al. (2016)  China | 3 experimental groups | n= 18  Sprague-Dawley rats  **Age:** adult  **Sex:** female  **Weight:** 220-250 g  **Comorbidities:** N/A | Traumatic SCI,  Crush injury compressing with a vascular clip (30g forces) for 2 min  **Level:** T9 | - Sham operated by laminectomy (n=6) - SCI + phenylbutyrate (PBA) 100 mg/kg i.p. administered immediately after SCI and then once a day for 2 weeks (n=6) - SCI + DMSO (vehicle) equal volume i.p. administered immediately after SCI and then once a day for 2 weeks (n=6) | BBB locomotor scale | Days 0, 1, 2, 3, 4, 5, 6, 7, 10, 14 post-SCI | one-way ANOVA and Dunnett’s post hoc test | - 4-PBA treatment significantly increased the hindlimb locomotor function 6 to 14 days after SCI, compared with vehicle-treated controls (p<0.01) |
| Wang et al. (2023) | 11 experimental groups | n= 66  C57BL/6 J mice  **Age:** adult  **Sex**: male  **Weight**: 20-30g  Comorbidities: N/A | Traumatic SCI  Contusion model using a computer-controlled impactor  **Level:** T10 | Experiment 2:   - SCI + SAHA 1 μg intrathecal administered 15 min before tests (n=6) - SCI + SAHA 3 μg intrathecal administered 15 min before tests (n=6) - SCI + SAHA 10 μg intrathecal administered 15 min before tests (n=6) - SCI + pregabalin 30 mg/kg i.p. 3 h before the tests after SCI (n=6)   Experiment 3:   - Sham operated by laminectomy (n=6) - SCI (n=6) - SCI + SAHA intrathecal administered 15 min before tests (n=6) dose of SAHA used was not specified by the authors   Experiment 4:   - SCI + DMSO + si-NC (adenovirus empty vector) intrathecal administered 15 min before tests (n=6) - SCI + SAHA 5μL intrathecal + si-NC intrathecal administered 15 min before tests (n=6) - SCI + DMSO + NEDD4 silencing adenovirus (si-NEDD4) intrathecal administered 15 min before tests (n=6) - SCI + SAHA 5μL intrathecal + si-NEDD4 intrathecal administered 15 min before tests (n=6)   In experiment 4 ach mouse was vertically injected with 20 μL si-NC or si-NEDD4 (1 × ­108 plaque-forming units) in the interval of L5-6 spinous processes with microinjector.  *Experiment 1 did not test an HDAC inhibitor hence was excluded | Experiment 2:   - Von Frey filament test - Thermal paw withdrawal latency test   Experiment 3, 4:   - The elevated plus maze test (EPMT) - Novelty suppressed feeding test (NSFT) - Forced swimming test (FST) - Open field test (OFT) | - **Von Frey filament test, thermal paw withdrawal latency test:** tested before SAHA treatment, at 15, 30, 45, 60, 90, 120, 180 min after SAHA treatment - **The elevated plus maze test, novelty suppressed feeding test, forced swimming test, open field test:** were all conducted 2 weeks after induction of SCI 60 min after SAHA administration | Experiment 2:   - **von Frey filament test, thermal paw withdrawal latency test:** two-way ANOVA and least significant difference test for post hoc multiple comparisons   Experiment 3:   - **for PFT, EPMT, NSFT, FST:** one-way ANOVA with Tukey's test to validate post hoc multiple comparisons   Experiment 4:   - **for PFT, EPMT, NSFT, FST:** one-way ANOVA with Tukey's test to validate post hoc multiple comparisons | - **Von Frey filament test:** after 10 µg of SAHA, there was no significant difference in mechanical threshold compared to pregabalin control group; there was significant increase in mechanical threshold after 10 µg of SAHA compared to before treatment (p<0.05); the effect of SAHA reached the peak value 60 min after drug administration, lasted till 90 min, and then vanished after 120 min - **Thermal paw withdrawal latency test:** there was no significant difference in reaction times between mice in the 10 µg of SAHA group and pregabalin-treated group; there was a significant increase in reaction times in µg SAHA-treated group compared to before treatment (p<0.05); the effect of SAHA reached the peak value 60 min after drug administration, lasted till 90 min, and then vanished after 120 min - **The elevated plus maze test:** there was an increase in the proportion of entries into the open arms as well as increase in the proportion of time spent in the open arms in the SAHA-treated SCI group compared to untreated SCI group (statistical significance was not reached); there was no significant difference in both parameters between the SAHA-treated SCI group and sham group - **Novelty suppressed feeding test:** eating latency was decreased after SAHA treatment of SCI mice compared to untreated SCI mice (statistical significance not reached); there was no significant difference in feeding latency in SAHA-treated group compared to sham group - **Forced swimming test**: the immobility time was reduced in the SAHA-treated SCI group compared to untreated SCI group (no statistical significance was reached); there was no difference in the total distance between the two groups; there was no significant difference in both above parameters between the SAHA-treated group and sham group - **Open field test:** there was an increase in number of entries to the center area as well as time in the center area compared to the untreated SCI group (statistical significance not reached); there was no significant difference in both parameters between SCI + SAHA group and sham group |
| Chu et al. (2015)  China | 3 experimental groups | n= 27  Sprague-Dawley rats  **Age:** adult  **Sex:** male  **Weight:** 180-220 g  **Comorbidities:** N/A | Traumatic SCI  Extradural compression of the spinal cord using an aneurysm clip at a closing force of 30g for 1 min  **Level:** T10 | - Sham operated by laminectomy (n=9) - SCI + saline i.p. every 12h from day 15 to day 22 after SCI (n=9) - SCI + VPA 150mg/kg i.p. every 12h from day 15 to day 22 after SCI (n=9) | BBB locomotor scale | every week for 8 weeks post-SCI | Two-way repeated measures ANOVA | - VPA-treated SCI group achieved significantly higher BBB score from 4 weeks to the end point compared to the saline-treated control (p<0.05) |
| Gao et al. (2020)  China | 4 experimental groups | n= 24  Sprague-Dawley rats  **Age:** not specified  Sex: male  **Weight:** 260-300g  **Comorbidities:** N/A | Traumatic SCI,  Contusion model using a 10g metal rod 2 mm diameter dropped from a height of 25 mm  Level: T9/10 | - Sham operated by laminectomy (n=6) - SCI + vehicle (saline, n=6) - SCI + melatonin (n=6) - SCI + melatonin + EX527 (n=6)   Melatonin was administered at a dose 10 mg/kg directly into the abdominal cavity after SCI and repeated at the same time on the 1^st^ and 2^nd^ day after SCI.  EX527 was administered at a dose 10 μg/kg directly into the abdominal cavity after SCI and at the same time on the 1^st^ and 2^nd^ day after SCI.  Saline was administered in the same way as melatonin and EX527. | BBB locomotor scale | Pre-SCI on day 0  Post-SCI on days 1, 3, 7, 14, 21, 28, 35, 42 | Mann–Whitney U test | - no significant difference in the BBB score was observed between the SCI + melatonin + EX527 compared to SCI + vehicle group (p>0.05) |
| Jiang et al. (2023)  China | 4 experimental groups | n= 40  C57BL/6J mice  **Age:** 8-week  **Sex**: female  **Weight**: 20-25g  **Comorbidities:** N/A | Traumatic SCI  Contusion model  using a 12.5g altered impactor (2mm diameter) dropped from a height of 15mm.  **Level:** T9-T10 | - Sham operated by laminectomy (n=10) - SCI + isotonic glucose i.p. 2h post-SCI once daily until day 3 post-SCI (n=10) - SCI + ZnG 30 mg/kg i.p. 2h post-SCI once daily until day 3 (n=10) - SCI + 3-TYP 50mg/kg i.p. 1 week before SCI, 1 day every other day, three times and post-SCI: 2 h postoperatively, once daily until day 3 (n=10) | Basso Mouse Scale | Pre-SCI assessed 1h before surgery  Post-SCI assessed on days 1, 3, 7, 14, 21, 28 | Non-parametric Kruskal­Wallis H test followed by Bonferroni correction | - The BMS score in 3-TYP-treated mice was not significantly different from that of the untreated SCI mice |
| Li et al. (2023)  China | 4 experimental groups | n=20  Sprague-Dawley rats  **Age**: 9-10 weeks old  **Sex:** female  **Weight**: 180-220g  **Comorbidities:** N/A | Traumatic SCI  Spinal cord compression vertically,  Using a sterile metal impounder of weight 35g, diameter 2mm for 5min  Level: T9-12 | - Sham operated by laminectomy (n=5) - SCI + vehicle (saline) i.p. immediately after the SCI (n=5) - SCI +OMT 40 mg/kg i.p. immediately after the SCI (n=5) - SCI + OMT + EX527, for EX527: 10 μg/kg i.p. immediately after SCI (n=5), the amount and route of administration for OMT was not specified by the authors for this group | - BBB locomotor scale - Footprint analysis | - **BBB score:** days 0,1,3,7 post-SCI - **Footprint analysis:** at 7 post-SCI | - **BBB:** two-factor ANOVA followed by Bonferroni post-hoc for repeated measures - **Footprint analysis:** no statistical analysis | - **BBB score:** beneficial effect of oxymatrine (putatively increases SIRT1 expression) after SCI was partly abrogated after administration of EX527 (p < 0.05) at day 7 - **Footprint analysis**: therapeutic effect of oxymatrine on gait was significantly inhibited by co-treatment with EX527 |
| Yu et al. (2019)  China | 6 experimental groups | n=36  Sprague-Dawley rats  **Age:** 8 weeks  **Sex:** male  **Weight:** 180-250g  **Comorbidities:** N/A | Ischemia/Reperfusion Injury (I/R)   The abdominal aorta was blocked above the right renal artery near the heart using a 50 g aneurysm clip for 60 min  **Level:** N/A | - Sham same procedure as I/R group but no occlusion of the aorta was performed (n=6) - I/R group + 0.9% saline i.p. immediately after reperfusion (n=6) - I/R + MLN4924 10 mg/kg (route of administration not specified), immediately after I/R injury (n=6) - I/R + MLN4924 30 mg/kg (route of administration not specified), immediately after I/R injury (n=6) - I/R + MLN4924 60 mg/kg (route of administration not specified), immediately after I/R injury (n=6) - I/R + MLN4924 30 mg/kg (route of administration not specified), immediately after I/R injury + EX527 1 mg/kg injected 0.5h before I/R injury (exact route of administration was not given) (n=6) | BBB locomotor scale | 1, 6, 12, 24h after reperfusion | One-way ANOVA | - the ability of MLN4924 to rescue neurological and motor function in SCI was reduced by EX527 (at 12h and 24h p<0.0001 compared between I/R +MLN4924 + EX527 group and I/R +MLN4924 group) |
| Cui et al. (2025)  China | 3 experimental groups | n=36  Sprague-Dawley rats  **Age:** not given  **Sex:** male  **Weight:** 220-240g  **Comorbidities:** N/A | Compression SCI model using an aneurysm clip to compress the right half of the spinal cord  **Level:** C5 | - sham group + saline - vertebral plate was removed without clamping the spinal cord (n=12) - SCI + saline (n=12) - SCI + sodium butyrate 300 mg/kg oral gavage once daily after SCI surgery, the duration of treatment was not given (n=12) | - cylinder rearing test - grooming test | Both tests were assessed 1 day before and 3-, 7-, 14-, and 28-days post-SCI | One-way analysis of variance with LSD (equal variances assumed). | - **Cylinder rearing test:** at 28 days post-SCI, the forelimb utilisation rate was significantly higher in the sodium butyrate group compared to untreated SCI group (p=0.013) - **Grooming test:** at 28 days post-SCI, the scores were significantly higher in the butyrate-treated group compared to the untreated SCI group (p=0.010) |
| Dai et al. (2024)  China | 4 experimental groups | n=24  Sprague-Dawley rats  **Age:** adult  **Sex:** female  **Weight:** 180-220g  **Comorbidities:** N/A | Traumatic SCI  Contusion model metal impactor 10 g, 2 mm diameter) dropped from a height of 50 mm  **Level:** T9-10 | - Sham operated by laminectomy (n=6) - SCI + vehicle (n=6) - SCI + ACY1215 25 mg/kg i.p. once weekly (n=6) - SCI + ACY1215 50 mg/kg i.p. once weekly (n=6) | BBB score | 0, 1, 7, 14, 21-, 28-, 35-, and 42-days post-SCI | One-way or two-way ANOVA with Holm– Sidak’s multiple comparisons test (for parametric data) and the Kruskal–Wallis test (for nonparametric data) | - In the high dose ACY1215 group (50mg/kg) there was statistically significant improvement in BBB scores from day 14 post-SCI compared to the untreated group (day 14: p<0.001; days 21, 28, 35, 42: p<0.0001) - In the low dose ACY1215 group (25mg/kg) there was statistically significant improvement in BBB scores from day 21 post-SCI compared to the untreated group (days 21, 42: p<0.01; days 28,35: p<0.001) |
| Kalimullina et al. (2024)  Canada, Switzerland | 4 experimental groups | n = 70  Wistar rats  **Age:** adult  **Sex:** male  **Weight:** 300g  **Comorbidities:** N/A | Traumatic SCI  Contusion model using an impactor device (2.5 mm impactor tip) 400kdyn, with a 5 s dwell time  **Level:** T3 | - SCI + vehicle (n=12) - SCI + fluoxetine i.p. 10 mg/kg either 1 h (n = 7) or 6 h (n = 10) after SCI and then every 12h for 2 weeks - SCI + glyburide i.p. 10μg/kg 1 h (n = 10) or 6 h (n = 9) after SCI and then every 12h for 2 weeks - SCI + VPA 300 mg/kg reduced to 150mg/kg later in experiment due to high mortality i.p. 1 h (n = 11) or 6 h (n = 11) after SCI and then every 12h for 2 weeks | BBB score | 1 week before and weekly after SCI for 4 weeks | Repeated measures two-way ANOVA with Tukey’s tests for multiple comparisons | - severe ulcerative gastritis and cystitis in the valproic acid group at 300 mg/kg dose which led to dose reduction to 150 mg/kg for the rest of the experiment - no neuroprotective drug had an impact on functional motor recovery of rodents following a severe T3 contusion |

**Table 5: Risk of bias assessment**

Systematic Review Center for Laboratory Animal Experimentation (SYRCLE) risk of bias assessment(1).

Each SYRCLE checklist question was scored as ‘Yes’, ‘No, or ‘Unclear’.

| **Bias assessment question** | Was the allocation sequence adequately generated and applied? (selection bias) | Were the groups similar at baseline or were they adjusted for confounders in the analysis? (selection bias) | Was the allocation adequately concealed? (selection bias) | Were the animals randomly housed during the experiment? (performance bias) | Were the caregivers and/or investigators blinded from knowledge which intervention each animal received during the experiment? (performance bias) | Were animals selected at random for outcome assessment? (detection bias) | Was the outcome assessor blinded? (detection bias) | Were incomplete outcome data adequately addressed? (attrition bias) | Are reports of the study free of selective outcome reporting? (reporting bias) | Was the study apparently free of other problems that could result in high risk of bias? (other sources of bias) |
| --- | --- | --- | --- | --- | --- | --- | --- | --- | --- | --- |
| Abdanipour et al. (2012) | Unclear | Unclear | Unclear | Unclear | Unclear | Unclear | Yes | Yes | Yes | No |
| Abematsu et al. (2010) | Unclear | Yes | Unclear | Unclear | Unclear | Unclear | Yes | Unclear | Yes | Yes |
| Chen et al. (2018) | Unclear | Yes | Unclear | Unclear | Unclear | Unclear | Yes | Yes | Yes | Yes |
| Chen et al. (2023) | Unclear | Yes | Unclear | Unclear | Unclear | Unclear | Yes | Yes | Yes | Yes |
| Dai et al. (2021) | Unclear | Yes | Unclear | Unclear | Unclear | Unclear | Unclear | Yes | Yes | Yes |
| Darvishi et al. (2014) | Unclear | Unclear | Unclear | Unclear | Unclear | Unclear | Unclear | Unclear | No | Yes |
| Gao et al. (2020) | Unclear | Unclear | Unclear | Unclear | Unclear | Unclear | Yes | Yes | Yes | Yes |
| Hao et al. (2013) | Unclear | Yes | Unclear | Unclear | Unclear | Unclear | Yes | Yes | Yes | Yes |
| He et al. (2017) | Unclear | Yes | Unclear | Unclear | Unclear | Unclear | Yes | Unclear | Yes | Yes |
| Hendrix et al. (2020) | Unclear | Unclear | Unclear | Unclear | Yes | Unclear | Yes | Unclear | Yes | No |
| Jafarimanesh et al. (2023) | Unclear | Unclear | Unclear | Unclear | Unclear | Unclear | Yes | Yes | No | Yes |
| Jiang et al. (2023) | Unclear | Yes | Unclear | Unclear | Unclear | Unclear | Yes | Unclear | Yes | No |
| Kuboyama et al. (2017) | Unclear | Unclear | Unclear | Unclear | Unclear | Unclear | Yes | Yes | Yes | Yes |
| Lanza et al. (2019) | Unclear | Yes | Unclear | Unclear | Unclear | Unclear | Yes | Yes | Yes | Yes |
| Lee et al. (2012) | Unclear | Yes | Unclear | Unclear | Unclear | Unclear | Yes | No | No | Yes |
| Lu et al. (2013) | Unclear | Yes | Unclear | Unclear | Unclear | Unclear | Yes | Unclear | Yes | Yes |
| Luo et al. (2023) | Unclear | Yes | Unclear | Unclear | Unclear | Unclear | Yes | Unclear | Yes | Yes |
| Lv et al. (2012) | Unclear | Unclear | Unclear | Unclear | Unclear | Unclear | Yes | Yes | Yes | Yes |
| Lv et al. (2011) | Unclear | Unclear | Unclear | Unclear | Unclear | Unclear | Yes | Yes | Yes | Yes |
| Mardi et al. (2021) | Unclear | Yes | Unclear | Unclear | Unclear | Unclear | Unclear | Yes | No | Yes |
| Mizukami et al. (2010) | Unclear | Unclear | Unclear | Unclear | Unclear | Unclear | Yes | Yes | Yes | No |
| Penas et al.  (2011) | Unclear | Yes | Unclear | Unclear | Unclear | Unclear | Yes | Yes | Unclear | Yes |
| Qi et al. (2018) | Unclear | Unclear | Unclear | Unclear | Unclear | Unclear | Yes | Yes | Yes | Yes |
| Qin et al. (2024) | Unclear | Unclear | Unclear | Unclear | Unclear | Unclear | Yes | Unclear | Yes | Yes |
| Reis et al. (2020) | Unclear | Yes | Unclear | Unclear | Unclear | Unclear | Unclear | Yes | Yes | Yes |
| Sanchez et al. (2018) | Unclear | Unclear | Unclear | Unclear | Unclear | Unclear | Yes | Unclear | Yes | Yes |
| Seira et al. (2020) | Unclear | Unclear | Unclear | Unclear | Unclear | Unclear | Unclear | Yes | Yes | Yes |
| Ulas et al. (2023) | Unclear | Unclear | Unclear | Unclear | Unclear | Unclear | Unclear | Yes | Yes | Yes |
| Wang et al. (2020) | Unclear | Yes | Unclear | Unclear | Unclear | Unclear | Unclear | Yes | Yes | Yes |
| Wang et al. (2021) | Unclear | Yes | Unclear | Unclear | Unclear | Unclear | Unclear | Yes | Yes | No |
| Yu et al. (2012) | Unclear | Yes | Yes | Unclear | Unclear | Unclear | Yes | Yes | Yes | Yes |
| Zhang et al. (2021) | Unclear | Yes | Unclear | Unclear | Unclear | Unclear | Unclear | Yes | Yes | No |
| Zhang et al. (2018) | Unclear | Unclear | Unclear | Unclear | Unclear | Unclear | Unclear | Unclear | Yes | Yes |
| Zheng et al. (2020) | Unclear | Yes | Unclear | Unclear | Unclear | Unclear | Yes | Yes | No | No |
| Zhou et al. (2016) | Unclear | Yes | Unclear | Unclear | Unclear | Unclear | Yes | Yes | Yes | Yes |
| Wang et al. (2023) | Unclear | Yes | Unclear | Unclear | Unclear | Unclear | Unclear | Yes | Yes | Yes |
| Chu et al. (2015) | Unclear | Yes | Unclear | Unclear | Unclear | Unclear | Yes | Yes | Yes | Yes |
| Li et al. (2023) | Unclear | Yes | Yes | Unclear | Unclear | Unclear | Unclear | Yes | Yes | Yes |
| Yu et al. (2019) | Yes | Yes | Unclear | Unclear | Unclear | Unclear | Yes | Yes | Yes | Yes |
| Cui et al. (2025) | Unclear | Unclear | Unclear | Unclear | Unclear | Unclear | Unclear | Yes | Yes | Yes |
| Dai et al. (2024) | Unclear | Yes | Unclear | Unclear | Unclear | Unclear | Yes | Yes | Yes | Yes |
| Kalimullina et al. (2024) | Unclear | Yes | Unclear | Unclear | Unclear | Unclear | Yes | Yes | Yes | No |

**Table 6: SWiM checklist(2).**

| **SWiM is intended to complement and be used as an extension to PRISMA** | |  |  |
| --- | --- | --- | --- |
| **SWiM reporting item** | **Item description** | **Page in manuscript where item is reported** | **Other*** |
| *Methods* | |  |  |
| **1** Grouping studies for synthesis | 1a) Provide a description of, and rationale for, the groups used in the synthesis (e.g., groupings of populations, interventions, outcomes, study design) | 6, 7 |  |
|  | 1b) Detail and provide rationale for any changes made subsequent to the protocol in the groups used in the synthesis | 6, 7 |  |
| **2** Describe the standardised metric and transformation methods used | Describe the standardised metric for each outcome. Explain why the metric(s) was chosen, and describe any methods used to transform the intervention effects, as reported in the study, to the standardised metric, citing any methodological guidance consulted | 6, 7 |  |
| **3** Describe the synthesis methods | Describe and justify the methods used to synthesise the effects for each outcome when it was not possible to undertake a meta-analysis of effect estimates | 6, 7 |  |
| **4** Criteria used to prioritise results for  summary and synthesis | Where applicable, provide the criteria used, with supporting justification, to select the particular studies, or a particular study, for the main synthesis or to draw conclusions from the synthesis (e.g., based on study design, risk of bias assessments, directness in relation to the review question) | 6, 7 |  |
| **5** Investigation of heterogeneity in reported effects | State the method(s) used to examine heterogeneity in reported effects when it was not possible to undertake a meta-analysis of effect estimates and its extensions to investigate heterogeneity | 6, 7 |  |
| **6** Certainty of evidence | Describe the methods used to assess certainty of the synthesis findings | 6, 7 |  |
| **7** Data presentation methods | Describe the graphical and tabular methods used to present the effects (e.g., tables, forest plots, harvest plots).  Specify key study characteristics (e.g., study design, risk of bias) used to order the studies, in the text and any tables or graphs, clearly referencing the studies included | 6, 7 |  |
| *Results* | | | |
| **8** Reporting results | For each comparison and outcome, provide a description of the synthesised findings, and the certainty of the findings. Describe the result in language that is consistent with the question the synthesis addresses, and indicate which studies contribute to the synthesis | 8-14 |  |
| *Discussion* | | | |
| **9** Limitations of the synthesis | Report the limitations of the synthesis methods used and/or the groupings used in the synthesis, and how these affect the conclusions that can be drawn in relation to the original review question | 17-18 |  |

PRISMA=Preferred Reporting Items for Systematic Reviews and Meta-Analyses.

**Table 7: Description of neurobehavioural outcome assessment methods used in the included**

**studies.**

| **Outcome assessment tool** | **Description** |
| --- | --- |
| **Locomotor function** | |
| Basso, Beattie, Bresnahan (BBB) Locomotor Score(3-29) | Assesses hindlimb movement and weight bearing, coordination of the hindlimb with the forelimbs, and placement of trunk and tail.  Graded from 0 – paralysis of the hindlimbs to 21 – normal hindlimb movement(30). |
| Basso Mouse Scale (BMS) (31-40)  and BMS sub-score(36) | Assesses hindlimb movements including ankle movements, paw placement, stepping pattern, coordination, trunk instability, and tail position, Graded from 0 - no movement of the hindlimbs to a maximum score of 9 - normal movement in the hindlimbs. For animals that achieved 5 or more in the BMS assessment, BMS sub-score may be calculated for a more thorough assessment of their locomotion(41). |
| Tarlov score(14, 42) | Assessed hindlimb movements. Graded from 0-4: 0 - no movement of the hindlimbs, 1 - perceptible movement of the hindlimb joints, 2 - good movement of the joints but unable to stand, 3 - able to stand and walk, 4 - complete recovery of hindlimb movement(42). |
| Inclined plane test(5, 10, 21, 38) | Determines the maximum angle at which an animal can maintain its grip on an inclined plane (up- and/or down-angled) for 5 seconds. It is measured from 0° to 90°(30). |
| Footprint analysis(12, 15, 16, 28, 39) | Series of sequential steps used to determine the mean values of limb rotation and of base of support(15). |
| Horizontal grid walk test(12, 38) | Assesses sensory-motor coordination of the limbs. Assesses the accuracy of paw placement and animal motor control required to cross a meter-long runaway made of horizontal round metal bars. Spaced unevenly to avoid habituation. Measures the number of foot falls(30). |
| Narrow beam test(16, 38) | Assesses descending motor control and body balance of animals.  Scored from 0 – complete inability to traverse the beam to 2 – normal weight support and accurate foot placement. If 3 different beams are used, scores for each can be summed up to give a maximum score of 6 points(43). |
| Horizontal ladder test(44) | Uses a horizontal ladder with spaced out ~ 50 rungs suspended above the ground. The scoring system is based on positive (plantar step/toe step/skip) and negative events (slip/miss/drag). Each rung is analysed and given a score from the aforementioned category. Ladder beam score (LBS %) is the number of positive events/total number of events and the cumulative number of errors (CE)(45). |
| Ladder walk test(38) | Uses a horizontal ladder with irregularly spaced rungs, suspended above the ground with walls on each side. Scoring system analyses each forepaw and hindpaw to give a compound score. Grading includes foot fault score, foot placement accuracy analysis and the forepaw digit score(46). |
| Grip strength(31) | Animals are given a chance to grasp a bar and are then gently pulled away from it parallel to the degree they grasped until they release the bar. The apparatus measures the grip force.  Score of 0 is given if the mouse cannot catch the crossbar(30). |
| Toyama Mouse Score (TMS)(34) | It is a modified score system with an emphasis on hindlimb body trunk support taking into consideration ankle movement, movement of knees, thighs, and toes. Graded on a scale of 0–30 points(34). |
| Cylinder test(44) | It is a rearing test which assesses forelimb preference during spontaneous  vertical exploration. Initial and subsequent use of the forepaw is scored over 10 rearing events(44). |
| **Pain** | |
| Von Frey filament test(40) | Used to establish the mechanical paw withdrawal threshold using innocuous mechanical stimulation. A von Frey filament is applied to the skin of the hindpaw or forepaw whilst looking for a withdrawal reaction. If no reaction is observed, then a higher force filament is used. The smallest filament that elicits a response is taken as the threshold stimulus(30). |
| Thermal paw withdrawal latency test(40) | Conducted on a 30 °C temperature-controlled glass platform, which generates a thermal stimulus to the surface of the outward hind paw. The paw withdrawal latency was the time from the start of stimulus to the moment when the paw stopped(40). |
| **Anxiety and depression-like behaviours** | |
| The elevated plus maze test(40) | The test is carried out in a maze made of two open arms and two closed arms. The assessed parameters included: entry number into and staying time in the open arms, as well as the total distance of movement(40). |
| Novelty suppressed feeding test(40) | Food is placed at the centre of the box and the animals are placed in the corner regions.  The result was the time from placing the mouse in the box to the moment it took its first bite of food(40). |
| Forced swimming test(40) | Animals are put in a cylinder filled with water and forced to swim for 6 minutes with the first minute used as the acclimation time. The time of immobility is recorded within the next 5 minutes. Immobility is defined as floating on the water or making subtle movement to keep the head out of the water(40). |
| Open field test(40) | The animal is placed in the central area of a box and its behaviour is recorded for 5 minutes. The assessed elements include the entry number into the central position, the staying time in the central position, and the overall distance(40). |

**Table 8: SCI models, advantages and limitations.** Six studies did not specify or reference the exact device used to induce SCI(6, 8, 22, 23, 28, 33) Adapted from Bhatti et al (2021)(47).

| **Type of SCI injury model** | **Description** | **Relevant methods** | **Advantages** | **Limitations** |
| --- | --- | --- | --- | --- |
| Contusion | Brief, acute injury to the spinal cord | - New York University (NYU) weight-drop device(3, 5, 7, 13, 15-17) - Modified NYU weight drop device(9) - Infinite Horizon impactor(4, 18, 19, 34, 39) - NYU impactor device(12) - Impact One Stereotaxic Impactor(40) - Modified Allen’s weight drop apparatus(25, 36) | - Allows control over the severity and location of induced SCI(48) - Depending on device used it may allow for real-time digital data recording(48) - Similar mechanism of injury to the majority of human SCI(49) | - Requires specialist equipment(47) - NYU weight drop device: ‘weight bounce’ effect may lead to multiple impacts causing variability between injured animals(48) - Infinite Horizon impactor: the included clamp system is suboptimal in stabilising the spinal column which may lead to significant displacement and variability in SCI(48) |
| Compression | Prolonged application of pressure on the spinal cord | - Microvascular clip(10, 24, 26, 27, 31, 35) - Dorsolateral funiculus crush(44) | - Microvascular clip: allows to adjust SCI severity by varying the clip closing force and/or application time(48) - Microvascular clip can be adapted to difference levels of the spinal cord: cervical, thoracic and lumbar(48) - Relatively cheap and simple to use(48) | - Microvascular clip: no measurable control over the force applied(48) - Does not involve the acute impact factor present in the contusion models(47) |
| Ischaemia/Reperfusion | Hypoperfusion of the spinal cord | - Ischaemia/Reperfusion via aortic clamping(14, 21, 29, 42) | - Useful for studying mechanism of secondary injury in SCI (inflammation and oxidative stress)(50) | - Complex pathophysiology resulting in less control over the degree of final SCI(51) - Variability in ischaemia/reperfusion response between species limiting translatability to human SCI(51) - Removes the primary phase of spinal cord injury limiting its application to studies of human SCI(47) |
| Transection (partial) | Partial cut within the spinal cord | - Hemisection of the spinal cord(11, 20, 32, 37, 38) | - Allows more precise targeting of desired anatomical locations(47) - Allows for comparison between injured and healthy neuronal fibres in one animal(48) | - Difficult to achieve consistent injury between animals (48) - This type of SCI mechanism is rarely seen in humans (52) |

**Table 9: Summary of included studies grouped by HDAC inhibitor.** Effect of HDAC inhibition on locomotor function assessed in each study. Adapted from Boon et al. (2021)(53).

| **Author**  **(Year)** | **Animal species used** | **Number of animals (in neurobehavioural analysis)** | **Injury model** | **Level of injury** | **HDAC inhibitor** | **Dose escalation study** | **Outcomes analysed** | **Time of assessment** | **Effect direction** |
| --- | --- | --- | --- | --- | --- | --- | --- | --- | --- |
| **Valproate (VPA)** | | | | | | | | | |
| Abdanipour et al. (2012) | Sprague-Dawley rats | 30 | Traumatic SCI,  Contusion model using a 10g metal rod with 2mm diameter dropped from a height of 25mm | T12-L1 | VPA | Yes | BBB locomotor score | Days 3, 7, 14, 21, 28 post-SCI | ▲ |
| Abematsu et al. (2010) | ICR mice | 46 | Traumatic SCI,  Contusion model using 90kdyn SCI device | T9-10 | VPA | No | - BBB locomotor score | From week 1 after SCI until week 14, assessed weekly | ◀ ▶ |
| Chen et al. (2018) | Wistar rats | 24 | Traumatic SCI,  Contusion model using a 10g metal rod dropped from a height of 25mm | T10 | VPA | No | - BBB locomotor score - Inclined plane test | BBB score and inclined plane test assessed on 1, 3, 7, 14 days post-SCI | ▲_2_ |
| Darvishi et al. (2014) | Sprague-Dawley rats | 200 | Traumatic SCI,  Contusion model using the NYU weight drop device with a 10g metal rod 2.5 mm diameter dropped from a height of 12.5mm | T8 | VPA | Yes | - BBB locomotor score | Daily 10 days pre-SCI and post-SCI on days 1, 3, 7 and then twice a week for 2 weeks and afterwards once a week for 12 weeks | ▲ |
| Hao et al. (2013) | Sprague-Dawley rats | 15 | Traumatic SCI,  Contusion model using modified New York University impactor with a 10g metal rod 2.5 mm diameter dropped from a height of 25 mm | T10 | VPA | No | - BBB locomotor score | Once a week for 6 weeks post-SCI | ▲ |
| Jafarimanesh et al. (2023) | Sprague-Dawley rats | 35 | Traumatic SCI  hemisection spinal cord injury | T9-10 | VPA | No | - BBB locomotor score | Pre-SCI and post-SCI on days 1, 3, 5, 7, 14, 28 | ▲ |
| Lee et al. (2012) | Sprague-Dawley rats | 30 | Traumatic SCI  Contusion model either moderate or moderately severe contusion using a 25 gm-cm or 50 gm-cm impactor device respectively | T9-10 | VPA | Yes | - BBB locomotor score - Horizontal grid walk test - Footprint analysis | **BBB score:** evaluated for 5 weeks after SCI, weekly  **Both horizontal grid walk test and footprint analysis:** at 35 days post-SCI | ▲_3_ |
| Lu et al. (2013) | Sprague-Dawley rats | 14 | Traumatic SCI  Contusion model  using a 10g rod dropped from a height of 50mm while keeping the dura intact | T9-10 | VPA | No | - BBB locomotor score | On days 3, 5, 7, 9, 12, 14, 16, 18, 21, 31 post-SCI | ▲ |
| Lv et al. (2012) | Wistar rats | 36 | Traumatic SCI  Contusion model  using an NYU impactor device to drop a 10g rod from a height of 12.5mm | T9 | VPA | No | - BBB locomotor score - Footprint analysis | **Both BBB score and footprint analysis**: for 6 weeks post-SCI, weekly | ▲_2_ |
| Lv et al. (2011) | Wistar rats | 36 | Traumatic SCI  Contusion model  using an NYU impactor device to drop a rod from a height of 12.5mm (weight of the rod not specified) | T8 | VPA | No | - BBB locomotor score - Narrow beam test - Footprint analysis | **BBB score:** after baseline testing, the animals were tested once a week for 8 weeks post-SCI  **Both narrow beam test and footprint analysis**: 8 weeks post-SCI | ▲_3_ |
| Mardi et al. (2021) | Wistar rats | 32 | Traumatic SCI  Severe contusion model  using a weight drop device | Not specified | VPA | No | - BBB locomotor score | Days 3, 7, 14, 21, 28 post-SCI | ▲ |
| Penas et al. (2011) | Sprague Dawley rats | 12 | Traumatic SCI  Contusion model  using the Infinite Horizon impactor device at 250 kDyn | T8 | VPA | No | - BBB locomotor score | Pre- SCI and post-SCI on days 3, 7, 14, 28, 35 | ▲ |
| Reis et al. (2020) | Wistar rats | 18 | Traumatic SCI  Right-side hemisection by placing a 28-gauge needle dorsi-ventrally at the midline of the cord and pulling it laterally to ensure a complete hemisection | T10 | VPA | No | - BBB locomotor score | 2 days post-SCI and then weekly until week 6 | ◀ ▶ |
| Ulas et al. (2023) | Wistar rats | 16 | Ischaemia/reperfusion injury  Cross-compression of the aorta using an  atraumatic microvascular clamp placed along the infrarenal and iliac bifurcation parts of the abdominal aorta for 45 minutes. | N/A | VPA | No | - BBB locomotor score - Inclined plane test | **Both BBB score and inclined plane test:** at 1^st^, 6^th^, 12th, 24^th^, 48th hour post-SCI | ▲_2_ |
| Wang et al. (2020) | Sprague-Dawley rats | 52 | Traumatic SCI  Contusion model established  using a weight drop apparatus, dropping a 5g rod at a vertical height of 80mm | T10 | VPA | No | - BBB locomotor score | Days 1, 3, 7, 14, 28 post-SCI | ▲ |
| Wang et al. (2021) | Sprague-Dawley rats | 45 | Traumatic SCI  Contusion model established  using a weight drop apparatus, dropping a 5g rod at a vertical height of 80mm | T10 | VPA | No | - BBB locomotor score | 1, 2, 3 and 4 weeks after SCI | ▲ |
| Yu et al. (2012) | Sprague-Dawley rats | 36 | Traumatic SCI,  Spinal cord compression with a modified aneurysm clip with a closing force of 30 grams, the clip was rapidly released from the applicator and applied vertically onto the exposed spinal cord for 2min | T9 | VPA | No | - BBB locomotor score | Each week for 2 weeks post-SCI | ▲ |
| Chu et al. (2015) | Sprague-Dawley rats | 27 | Traumatic SCI  Extradural compression of the spinal cord using an aneurysm clip at a closing force of 30g for 1 min | T10 | VPA | No | - BBB locomotor score | Every week for 8 weeks post-SCI | ▲ |
| Hendrix et al. (2020) | Balb/c mice | 21-27*  *unclear | Traumatic SCI  T-cut spinal cord hemisection injury using iridectomy scissors | T8 | - PCI-34051 - VPA | No | - BMS score | Every day for first 8 days post-SCI, then very other day until day 35 post-SCI | ◀ ▶ |
| Kalimullina et al. (2024) | Wistar rats | 70*  *unclear | Traumatic SCI  Contusion model using an impactor device (2.5 mm impactor tip) 400kdyn, with a 5 s dwell time | T3 | VPA | No | - BBB locomotor score | 1 week before and weekly after SCI for 4 weeks | ◀ ▶ |
| **4-phenylbutyrate (4-PBA)** | | | | | | | | | |
| He et al. (2017) | Sprague-Dawley rats | 32 | Traumatic SCI,  Compression of spinal cord with a vascular clip (15 g) for 2 minutes | T9 | 4-PBA | No | - BBB locomotor score - Inclined plane test | Both BBB locomotor scale and inclined plane test assessed on days 1, 3, 7, 14, 28 post-SCI | ▲_2_ |
| Luo et al. (2023) | Sprague-Dawley rats | 25 | Ischemia/Reperfusion Injury (I/R)  Aortic clamping between the left and right renal arteries with a non-invasive artery clamp for 85 minutes. | N/A | 4-PBA | No | - BBB locomotor score - Tarlov scoring system | **Both BBB score and Tarlov score:** assessed over 3 min post-reperfusion for 4h | ▲_2_ |
| Zhang et al. (2021) | Sprague-Dawley rats | 24 | Traumatic SCI  Contusion model established  using a modified Allen’s weight drop apparatus, dropping a 10g rod at a vertical height of 25mm | T10 | 4-PBA | No | - BBB locomotor score | Day 7 post-SCI | ▲ |
| Zhou et al. (2016) | Sprague-Dawley rats | 18 | Traumatic SCI,  Crush injury compressing with a vascular clip (30g forces) for 2 min | T9 | 4-PBA | No | - BBB locomotor score | Days 0, 1, 2, 3, 4, 5, 6, 7, 10, 14 post-SCI | ▲ |
| Lanza et al. (2019) | CD1 mice | 50 | Traumatic SCI,  Extradural compression with aneurysm clip (24 g) for 1 minute. | T6-7 | 4-PBA | Yes | - BMS score | Assessed daily until day 9 | ▲ |
| Mizukami et al. (2010) | Japanese white rabbits | 18 | Ischemia/Reperfusion Injury (I/R),  the abdominal aorta just distal to the left renal artery and just proximal to the iliac bifurcation was cross-clamped and isolated for 15min to produce spinal cord ischaemia | NA | 4-PBA | No | - Tarlov score | Assessed at 8 hours, and 2 and 7 days after reperfusion | ▲ |
| Cui et al. (2025) | Sprague-Dawley rats | 36 | Compression SCI model using an aneurysm clip to compress the right half of the spinal cord | C5 | 4-PBA | No | - cylinder rearing test - grooming test | Both tests were assessed 1 day before and 3-, 7-, 14-, and 28-days post-SCI | ▲_2_ |
| **Class I HDAC inhibitors (RGFP966, Entinostat, CI-994, PCI-34051)** | | | | | | | | | |
| Chen et al. (2023) | Sprague-Dawley rats | 24 | Traumatic SCI,  Contusion model using a 10g metal rod dropped from a height of 25mm | T10 | RGFP966 | No | - BBB locomotor score | Days 1, 3, 7, 14 post-SCI | ▲ |
| Kuboyama et al. (2017) | C57BL/6J mice | 16 | Contusion injury using an infinite horizon impactor 70 kDyn force | T8 | RGFP966 | No | - BMS score - Toyama mouse score (TMS) | Both BMS and TMS: assessed every day for 30 days post-SCI | ▲_2_ |
| Sanchez et al. (2018) | Balb/c mice | 54-69*  *unclear | Traumatic SCI,  T-cut spinal cord hemisection injury using iridectomy scissors to transect left and right dorsal funiculus, the dorsal horns and the ventral funiculus. | T8 | - RGFP966 - scriptaid | No | - BMS score | From 1 day post-SCI, in the first week scored daily and then every other day until day 27 post-SCI | ◀ ▶ |
| Dai et al. (2021) | C57BL/6 mice | 40 | Traumatic SCI,  epidural compression of the spinal cord with a 24-g closure force applied for 1 min | T6-T7 | Entinostat | Yes | - BMS score - Forelimb grip strength | **Both BMS and forelimb grip strength:** assessed pre-SCI and post-SCI on days 1, 2, 3, 7, 14, 21, 28 | ▲_2_ |
| Zhang et al. (2018) | C57BL/6 J mice | 43 | Traumatic SCI,  Dorsal hemi-section using a surgical blade at a depth of 1.0 mm. Avoiding the remaining portion of the lateral CST, the surgical blade was passed through the dorsal spinal cord several times to create a lesion that extended downward to the central canal. | T8 | CI-994 | No | - BMS score - narrow beam walk test - horizontal grid walk test - ladder walk test - inclined plane test | **BMS:**assessed just before surgery and post-SCI at 1, 3, 7, 14, 21, 28, 35, 42  **Narrow beam walk test:** assessed just before surgery and post-SCI on days 7, 14, 21, 28, 35, 42  **Horizontal grid walk test:** assessed just before surgery and post-SCI on days 14, 21, 28, 35, 42  **Ladder walk test:** tests started 2 weeks post-SCI and continued once a week for additional 4 weeks  **Inclined plane test:** assessed pre-SCI, and post-SCI on days 7, 14, 21, 28, 35, 42 | ▲_5_ |
| Hendrix et al. (2020) | Balb/c mice | 21-27*  *unclear | Traumatic SCI  T-cut spinal cord hemisection injury using iridectomy scissors | T8 | - PCI-34051 - VPA | No | - BMS score | Every day for first 8 days post-SCI, then very other day until day 35 post-SCI | ◀ ▶ |
| **Class IIb inhibitors (Tubastatin A, SW-100, ACY1215)** | | | | | | | | | |
| Zheng et al. (2020) | C57BL/6OlaCnc mice | 50 | Traumatic SCI  Contusion model established  By dropping a 10g weight at a vertical height of 15mm | T10 | Tubastatin A | No | - BMS score - Footprint analysis | **BMS and footprint analysis**: assessed post-SCI on days 0, 1, 3, 7, 14, 28 | ▲_2_ |
| Qin et al. (2024) | not specified | 15 | Traumatic SCI  Contusion model  using modified Allen’s weight drop apparatus, dropping a 10g weight at a vertical height of 25mm | T10 | SW-100 | No | - BMS score | Before SCI, immediately after, and on days 1, 3, 7, 14, 21, 28 post-SCI | ▲ |
| Dai et al. (2024) | Sprague-Dawley rats | 24 | Traumatic SCI  Contusion model metal impactor 10 g, 2 mm diameter) dropped from a height of 50 mm | T9-10 | ACY1215 | Yes | - BBB locomotor score | 0, 1, 7, 14, 21-, 28-, 35-, and 42-days post-SCI | ▲ |
| **Class III inhibitors (EX527, 3-TYP)** | | | | | | | | | |
| Gao et al. (2020) | Sprague-Dawley rats | 24 | Traumatic SCI,  Contusion model using a 10g metal rod 2 mm diameter dropped from a height of 25 mm | T9-10 | EX527 | No | - BBB locomotor score | Days 0, 1, 3, 7, 14, 21, 28, 35, 42 post-SCI | ◀ ▶ |
| Li et al. (2023) | Sprague-Dawley rats | 20 | Traumatic SCI  Spinal cord compression vertically,  Using a sterile metal impounder of weight 35g, diameter 2mm for 5min | T9-12 | EX527 | No | - BBB locomotor score - Footprint analysis | **BBB score:** days 0,1,3,7 post-SCI  **Footprint analysis:** at 7 post-SCI | ◀ ▶_2_ |
| Yu et al. (2019) | Sprague-Dawley rats | 36 | Ischemia/Reperfusion Injury (I/R). The abdominal aorta was blocked above the right renal artery near the heart using a 50 g aneurysm clip for 60 min | N/A | EX527 | No | - BBB locomotor score | 1, 6, 12, 24h after reperfusion | ▼ |
| Jiang et al. (2023) | C57BL/6J mice | 40 | Traumatic SCI  Contusion model  using a 12.5g altered impactor (2mm diameter) dropped from a height of 15mm. | T9-10 | 3-TYP | No | - BMS score | Assessed pre-SCI, 1h before surgery and post-SCI on days 1, 3, 7, 14, 21, 28 | ◀ ▶ |
| **Other HDAC inhibitors (Trichostatin A, TMP269, Scriptaid)** | | | | | | | | | |
| Qi et al. (2018) | C57BL/6J mice | 30 | Traumatic SCI  Contusion model  using the Infinite Horizon impactor device at 60 kDyn | T9-10 | TMP269 | No | - BBB locomotor score | Twice daily for the first week post-SCI and weekly afterwards until 6 weeks post-SCI | ▼ |
| Seira et al. (2020) | C57Bl6 Pten floxed mice | 28 | Traumatic SCI,  Dorsolateral funiculus (DLF) crush with  fine forceps modified for this purpose by grinding their blades to a width of ~200 mm, one of the prongs was inserted into the dorsal horn gray matter (~1 mm deep) while the other prong was on the lateral surface of the spinal cord. The forceps were closed and held for 15 seconds to crush the dorsolateral funiculus containing the RST (and this was repeated once). | C4-5 | Trichostatin A (TSA) | No | - Cylinder test - Horizontal ladder test | **Cylinder test:** assessed post-SCI on days 3, 8, 15, 28 and 8 and 12 weeks post-SCI  **Horizontal ladder test:** assessed pre-SCI to obtain preinjury scores and then post-SCI on days 3, 8, 15, 28 and 8 and 12 weeks post-SCI | ◀ ▶_2_ |
| Sanchez et al. (2018) | Balb/c mice | 54-69*  *unclear | Traumatic SCI,  T-cut spinal cord hemisection injury using iridectomy scissors to transect left and right dorsal funiculus, the dorsal horns and the ventral funiculus. | T8 | - RGFP966 - scriptaid | No | - BMS score | From 1 day post-SCI, in the first week scored daily and then every other day until day 27 post-SCI | ◀ ▶ |

Figure legend:

▲ – positive direction of effect

▼ – negative direction of effect

◀ ▶ - no clear effect/conflicting findings

Number indicates the number of neurobehavioural outcomes which were reported for locomotor function domain.

**Table 10: Proposed mechanism of action of HDAC inhibitors.**

| **HDAC inhibitor** | **Mechanism of action** |
| --- | --- |
| Valproate | **Anti-apoptotic properties:**   - inhibits expression of pro-apoptotic factors: cleaved caspase-3 and Bax and increases expression of anti-apoptotic factors such as Bcl-2 in vivo(5) - reduces the apoptosis rate partly through decreased expression of Mst1 (cytoplasmic protein which downstream targets promote apoptosis (JNK/p28 and FOXO)(17)   **Anti-inflammatory properties:**   - promotes a phenotypic shift in microglial/macrophage phenotype from the M1-like (pro-inflammatory) to M2-like (anti-inflammatory). This is thought to be the reverse of the process happening after SCI(5) - reduces expression of pro-inflammatory cytokines such as TNF-α, IL-1β, IFN-γ(5) - increases acetylation of STAT1 which in turn increases the formation of STAT1-NF-κB p65 complexes which prevents NF-κB p65 from translocating to the nucleus, inhibiting its transcriptional activity(5)   **Anti-oxidative properties:**   - increases gene expression of antioxidative enzymes such as catalase and glutathione peroxidase as well as the total antioxidant status which could limit the amount of reactive oxygen species at the SCI site(21)   **Neurogenesis:**   - increases the expression of BDNF and GDNF neurotrophic factors in vitro(54) - it may protect against the inhibitory environment for neuronal growth created by Nogo-A(15) |
| 4-PBA | - inhibits the endoplasmatic reticulum stress response, acts as a chemical chaperone and aids protein folding which in turn reduces ER stress(42) - ER stress contributes to the activation of apoptosis and 4-PBA treatment appears to reduce neuronal apoptosis following ischaemia/reperfusion injury. Furthermore, the effect of 4-PBA is sustained through both the immediate and delayed phases of spinal cord injury due to ischaemia(42) |
| CI-994 | - inhibits neutrophil accumulation at the spinal cord injury site which reduces pro-inflammatory cytokine (e.g. TNF-α) activation and promotes neuronal growth around the lesion site(38) |
| Entinostat | - reduced NF-κB expression and nuclear translocation as well as reduced the levels of pro-inflammatory cytokines TNF-α and IL-1β postoperatively in the hippocampus of rats which is similar to valproate effect in the spinal cord(55) - may have anti-apoptotic effects through inhibition of the NLRP3 inflammasome(31) |
| PCI-34051 | - it may inhibit MMP9 (thought to be involved in blood-spinal cord barrier (BSCB) damage and increased permeability following the SCI) which could decrease the microglia/macrophage presence at the lesion site(32) - does not seem to affect the microglial phenotype(32) |
| RGFP966 | - reduces microglial activation(6, 56) - reduces the number of pro-inflammatory cytokines (TNF-α, IL-1β, IL-6)(6, 56) - promotes the shift from the M1-like macrophage phenotype to M2-like phenotype(37) |
| Scriptaid | - additional inhibition of HDAC1 may shift the macrophage polarisation towards the M1-like phenotype balancing out the M2-like phenotype shift which appears to be mediated by HDAC3 inhibition(37) |
| SW-100 | - increases the stability of microtubules and activates autophagy promoting neurite outgrowth(36) - increases the amount of acetylated α-tubulin which as a result promotes antero- and retrograde transport in neurones allowing for timely delivery of synaptic proteins and removal of damaged organelles at the synaptic ends in vitro(57) |
| TMP269 | - has an opposite effect to class I and class IIb HDAC inhibitors and promotes production of pro-inflammatory cytokines in macrophages after inflammatory stimulation in vitro and in vivo(19) - supports a shift in macrophage polarisation towards the M1-like phenotype(19) |
| Trichostatin A | - increased BDNF expression in primary rat cortical neurones after an ischaemic insult(58) |
| Tubastatin A | - increased tubulin acetylation supporting motor protein recruitment and allowing for better retrograde transport in neurones, particularly important for autophagy(39) |
| ACY1215 | - selective HDAC6 inhibitor - inhibits the NF-κB and STAT3 signalling pathways which contributes to its anti-inflammatory and neuroprotective effects(59) |
| Vorinostat | - may reduce expression of the Nav1.7 channel (important in pain sensation) by upregulating the ubiquitin ligase NEDD4 that in turn targets Nav1.7 channels for degradation(40) |
| 3-TYP | - has pro-oxidative effects and decreases mitophagy by opposing the action of Sirtuin 3(33) |
| EX527 | - Sirtuin 1 activation rather than inhibition seems to have a neuroprotective effect following SCI in animal models(60) - Sirtuin 1 appears to increase autophagy via activation of AMPK and one of its effects is an improvement in autophagosome-lysosome fusion(8, 28) - the Sirt1/AMPK pathway appears to inhibit apoptosis which may further contribute to the neuroprotective effects of sirtuin1 activation following SCI(8, 28) |

**Table 11:** **FDA-approved HDAC inhibitors.**

| **HDAC inhibitor** | **Indication** |
| --- | --- |
| Valproate | - epilepsy with partial seizures, absence seizures, and multiple seizure types(61) - bipolar disorder(61) |
| 4-PBA | - adjunct therapy in urea cycle disorders(62) |
| Vorinostat | - advanced primary cutaneous T-cell lymphoma(63) |
| Panobinostat | - multiple myeloma |
| Belinostat | - relapsed or refractory peripheral T-cell lymphoma |
| Romidepsin | - cutaneous T-cell lymphoma for patients who have received at least one prior systemic treatment |

1. Hooijmans CR, Rovers MM, de Vries RBM, Leenaars M, Ritskes-Hoitinga M, Langendam MW. SYRCLE’s risk of bias tool for animal studies. BMC Medical Research Methodology. 2014;14(1):43.

2. Campbell M, McKenzie JE, Sowden A, Katikireddi SV, Brennan SE, Ellis S, et al. Synthesis without meta-analysis (SWiM) in systematic reviews: reporting guideline. BMJ. 2020;368:l6890.

3. Abdanipour A, Schluesener HJ, Tiraihi T. Effects of valproic acid, a histone deacetylase inhibitor, on improvement of locomotor function in rat spinal cord injury based on epigenetic science. Iran. 2012;16(2):90-100.

4. Abematsu M, Tsujimura K, Yamano M, Saito M, Kohno K, Kohyama J, et al. Neurons derived from transplanted neural stem cells restore disrupted neuronal circuitry in a mouse model of spinal cord injury. J Clin Invest. 2010;120(9):3255-66.

5. Chen S, Ye J, Chen X, Shi J, Wu W, Lin W, et al. Valproic acid attenuates traumatic spinal cord injury-induced inflammation via STAT1 and NF-kappaB pathway dependent of HDAC3. J Neuroinflammation. 2018;15(1):150.

6. Chen S, Ye J, Wu G, Shi J, Li X, Chen X, et al. Histone Deacetylase 3 Inhibition Ameliorates Microglia-Mediated Neuro-Inflammation Via the SIRT1/Nrf2 Pathway After Traumatic Spinal Cord Injury. Neurorehabil Neural Repair. 2023;37(8):503-18.

7. Darvishi M, Tiraihi T, Mesbah-Namin SA, Delshad A, Taheri T. Decreased GFAP expression and improved functional recovery in contused spinal cord of rats following valproic acid therapy. Neurochemical Research. 2014;39(12):2319-33.

8. Gao K, Niu J, Dang X. Neuroprotection of melatonin on spinal cord injury by activating autophagy and inhibiting apoptosis via SIRT1/AMPK signaling pathway. Biotechnol Lett. 2020;42(10):2059-69.

9. Hao HH, Wang L, Guo ZJ, Bai L, Zhang RP, Shuang WB, et al. Valproic acid reduces autophagy and promotes functional recovery after spinal cord injury in rats. Neurosci Bull. 2013;29(4):484-92.

10. He Z, Zou S, Yin J, Gao Z, Liu Y, Wu Y, et al. Inhibition of Endoplasmic Reticulum Stress Preserves the Integrity of Blood-Spinal Cord Barrier in Diabetic Rats Subjected to Spinal Cord Injury. Scientific Reports. 2017;7(1):7661.

11. Jafarimanesh MA, Ai J, Shojaei S, Khonakdar HA, Darbemamieh G, Shirian S. Sustained release of valproic acid loaded on chitosan nanoparticles within hybrid of alginate/chitosan hydrogel with/without stem cells in regeneration of spinal cord injury. Prog. 2023;12(2):75-86.

12. Lee JY, Kim HS, Choi HY, Oh TH, Ju BG, Yune TY. Valproic acid attenuates blood-spinal cord barrier disruption by inhibiting matrix metalloprotease-9 activity and improves functional recovery after spinal cord injury. Journal of Neurochemistry. 2012;121(5):818-29.

13. Lu WH, Wang CY, Chen PS, Wang JW, Chuang DM, Yang CS, et al. Valproic acid attenuates microgliosis in injured spinal cord and purinergic P2X4 receptor expression in activated microglia. J Neurosci Res. 2013;91(5):694-705.

14. Luo L, Wang Y, Tong J, Li L, Zhu Y, Jin M. Xenon postconditioning attenuates neuronal injury after spinal cord ischemia/reperfusion injury by targeting endoplasmic reticulum stress-associated apoptosis. Neurosurg Rev. 2023;46(1):213.

15. Lv L, Han X, Sun Y, Wang X, Dong Q. Valproic acid improves locomotion in vivo after SCI and axonal growth of neurons in vitro. Experimental Neurology. 2012;233(2):783-90.

16. Lv L, Sun Y, Han X, Xu CC, Tang YP, Dong Q. Valproic acid improves outcome after rodent spinal cord injury: potential roles of histone deacetylase inhibition. Brain Res. 2011;1396:60-8.

17. Mardi A, Biglar A, Nejatbakhsh R, Abdanipour A. Valproic Acid Ameliorates Locomotor Function in the Rat Model of Contusion via Alteration of Mst1, Bcl-2, and Nrf2 Gene Expression. Iran. 2021;25(4):303-7.

18. Penas C, Verdu E, Asensio-Pinilla E, Guzman-Lenis MS, Herrando-Grabulosa M, Navarro X, et al. Valproate reduces CHOP levels and preserves oligodendrocytes and axons after spinal cord injury. Neuroscience. 2011;178:33-44.

19. Qi X, Wang P. Class IIa HDACs inhibitor TMP269 promotes M1 polarization of macrophages after spinal cord injury. J Cell Biochem. 2018;119(4):3081-90.

20. Reis KP, Sperling LE, Teixeira C, Sommer L, Colombo M, Koester LS, et al. VPA/PLGA microfibers produced by coaxial electrospinning for the treatment of central nervous system injury. Braz J Med Biol Res. 2020;53(4):e8993.

21. Ulas M, Argadal OG. Trace element, antioxidant and oxidant levels in spinal cord injury: different perspective on the effects of valproic acid. Eur Rev Med Pharmacol Sci. 2023;27(9):3892-905.

22. Wang D, Wang K, Liu Z, Wang Z, Wu H. Valproic acid-labeled chitosan nanoparticles promote recovery of neuronal injury after spinal cord injury. Aging (Albany NY). 2020;12(10):8953-67.

23. Wang D, Wang K, Liu Z, Wang Z, Wu H. Valproic Acid Labeled Chitosan Nanoparticles Promote the Proliferation and Differentiation of Neural Stem Cells After Spinal Cord Injury. Neurotox Res. 2021;39(2):456-66.

24. Yu SH, Cho DC, Kim KT, Nam KH, Cho HJ, Sung JK. The neuroprotective effect of treatment of valproic Acid in acute spinal cord injury. J. 2012;51(4):191-8.

25. Zhang H, Piao M, Guo M, Meng L, Yu H. MicroRNA-211-5p attenuates spinal cord injury via targeting of activating transcription factor 6. Tissue Cell. 2021;68:101459.

26. Zhou Y, Ye L, Zheng B, Zhu S, Shi H, Zhang H, et al. Phenylbutyrate prevents disruption of blood-spinal cord barrier by inhibiting endoplasmic reticulum stress after spinal cord injury. Am J Transl Res. 2016;8(4):1864-75.

27. Chu W, Yuan J, Huang L, Xiang X, Zhu H, Chen F, et al. Valproic Acid Arrests Proliferation but Promotes Neuronal Differentiation of Adult Spinal NSPCs from SCI Rats. Neurochemical Research. 2015;40(7):1472-86.

28. Li J, Cao Y, Li LN, Chu X, Wang YS, Cai JJ, et al. Neuroprotective Effects of Oxymatrine via Triggering Autophagy and Inhibiting Apoptosis Following Spinal Cord Injury in Rats. Mol Neurobiol. 2023;60(8):4450-71.

29. Yu S, Xie L, Liu Z, Li C, Liang Y. MLN4924 Exerts a Neuroprotective Effect against Oxidative Stress via Sirt1 in Spinal Cord Ischemia-Reperfusion Injury. Oxid Med Cell Longev. 2019;2019:7283639.

30. Tetreault LA, Zhu MP, Wilson JR, Karadimas SK, Fehlings MA-O. The Impact of Riluzole on Neurobehavioral Outcomes in Preclinical Models of Traumatic and Nontraumatic Spinal Cord Injury: Results From a Systematic Review of the Literature. (2192-5682 (Print)).

31. Dai C, Liu B, Peng B, Qu B, Lin J, Peng B, et al. Entinostat Improves Motor Function and Neuronal Damage Via Downregulating NLRP3 Inflammasome Activation After Spinal Cord Injury. Front Pharmacol. 2021;12:774539.

32. Hendrix S, Sanchez S, Ventriglia E, Lemmens S. HDAC8 Inhibition Reduces Lesional Iba-1+ Cell Infiltration after Spinal Cord Injury without Effects on Functional Recovery. Int. 2020;21(12):25.

33. Jiang D, Yang X, Ge M, Hu H, Xu C, Wen S, et al. Zinc defends against Parthanatos and promotes functional recovery after spinal cord injury through SIRT3-mediated anti-oxidative stress and mitophagy. CNS Neurosci Ther. 2023;29(10):2857-72.

34. Kuboyama T, Wahane S, Huang Y, Zhou X, Wong JK, Koemeter-Cox A, et al. HDAC3 inhibition ameliorates spinal cord injury by immunomodulation. Scientific reports. 2017;7(1):8641.

35. Lanza M, Campolo M, Casili G, Filippone A, Paterniti I, Cuzzocrea S, et al. Sodium Butyrate Exerts Neuroprotective Effects in Spinal Cord Injury. Mol Neurobiol. 2019;56(6):3937-47.

36. Qin T, Li C, Xu Y, Qin Y, Jin Y, He R, et al. Local delivery of EGFR+NSCs-derived exosomes promotes neural regeneration post spinal cord injury via miR-34a-5p/HDAC6 pathway. Bioactive Materials. 2024;33:424-43.

37. Sanchez S, Lemmens S, Baeten P, Sommer D, Dooley D, Hendrix S, et al. HDAC3 inhibition promotes alternative activation of macrophages but does not affect functional recovery after spinal cord injury. Experimental Neurobiology. 2018;27(5):437-52.

38. Zhang S, Fujita Y, Matsuzaki R, Yamashita T. Class i histone deacetylase (HDAC) inhibitor CI-994 promotes functional recovery following spinal cord injury article /13/51 /64/60 /38/77. Cell Death and Disease. 2018;9(5):460.

39. Zheng Z, Zhou Y, Ye L, Lu Q, Zhang K, Zhang J, et al. Histone deacetylase 6 inhibition restores autophagic flux to promote functional recovery after spinal cord injury. Experimental Neurology. 2020;324:113138.

40. Wang C, Chen R, Zhu X, Zhang X. Suberoylanilide Hydroxamic Acid Ameliorates Pain Sensitization in Central Neuropathic Pain After Spinal Cord Injury via the HDAC5/NEDD4/SCN9A Axis. Neurochemical Research. 2023;48(8):2436-50.

41. Leibinger M, Zeitler C, Gobrecht P, Andreadaki A, Gisselmann G, Fischer D. Transneuronal delivery of hyper-interleukin-6 enables functional recovery after severe spinal cord injury in mice. Nature Communications. 2021;12(1):391.

42. Mizukami T, Orihashi K, Herlambang B, Takahashi S, Hamaishi M, Okada K, et al. Sodium 4-phenylbutyrate protects against spinal cord ischemia by inhibition of endoplasmic reticulum stress. J Vasc Surg. 2010;52(6):1580-6.

43. Metz GAS, Merkler D, Dietz V, Schwab ME, Fouad K. Efficient testing of motor function in spinal cord injured rats. Brain Research. 2000;883(2):165-77.

44. Seira O, Wang W, Lee S, Roskams J, Tetzlaff W. HDAC inhibition leads to age-dependent opposite regenerative effect upon PTEN deletion in rubrospinal axons after SCI. Neurobiol Aging. 2020;90:99-109.

45. Cummings BJ, Engesser-Cesar C, Cadena G, Anderson AJ. Adaptation of a ladder beam walking task to assess locomotor recovery in mice following spinal cord injury. Behavioural Brain Research. 2007;177(2):232-41.

46. Metz GA, Whishaw IQ. Cortical and subcortical lesions impair skilled walking in the ladder rung walking test: a new task to evaluate fore- and hindlimb stepping, placing, and co-ordination. Journal of Neuroscience Methods. 2002;115(2):169-79.

47. Bhatti FI, Mowforth OD, Butler MB, Bhatti AI, Adeeko S, Akhbari M, et al. Systematic review of the impact of cannabinoids on neurobehavioral outcomes in preclinical models of traumatic and nontraumatic spinal cord injury. Spinal Cord. 2021;59(12):1221-39.

48. Cheriyan T, Ryan DJ, Weinreb JH, Cheriyan J, Paul JC, Lafage V, et al. Spinal cord injury models: a review. Spinal Cord. 2014;52(8):588-95.

49. Nobunaga AI, Go Bk Fau - Karunas RB, Karunas RB. Recent demographic and injury trends in people served by the Model Spinal Cord Injury Care Systems. (0003-9993 (Print)).

50. Castillo RL, González-Candia A, Carrasco R. Editorial: Mechanisms of Ischemia-Reperfusion Injury in Animal Models and Clinical Conditions: Current Concepts of Pharmacological Strategies. Frontiers in Physiology. 2022;13.

51. Soares RA-O, Losada DM, Jordani MC, Évora P, Castro ESO. Ischemia/Reperfusion Injury Revisited: An Overview of the Latest Pharmacological Strategies. LID - 10.3390/ijms20205034 [doi] LID - 5034. (1422-0067 (Electronic)).

52. Kundi S, Bicknell R, Ahmed Z. Spinal Cord Injury: current Mammalian Models. American Journal of Neuroscience. 2013;4:1-12.

53. Boon MA-O, Thomson H. The effect direction plot revisited: Application of the 2019 Cochrane Handbook guidance on alternative synthesis methods. (1759-2887 (Electronic)).

54. Wu X, Chen Ps Fau - Dallas S, Dallas S Fau - Wilson B, Wilson B Fau - Block ML, Block Ml Fau - Wang C-C, Wang Cc Fau - Kinyamu H, et al. Histone deacetylase inhibitors up-regulate astrocyte GDNF and BDNF gene transcription and protect dopaminergic neurons. (1461-1457 (Print)).

55. Wu Y, Dou J, Wan X, Leng Y, Liu X, Chen L, et al. Histone Deacetylase Inhibitor MS-275 Alleviates Postoperative Cognitive Dysfunction in Rats by Inhibiting Hippocampal Neuroinflammation. (1873-7544 (Electronic)).

56. Yang R, Song C, Chen J, Zhou L, Jiang X, Cao X, et al. Limonin ameliorates acetaminophen-induced hepatotoxicity by activating Nrf2 antioxidative pathway and inhibiting NF-κB inflammatory response via upregulating Sirt1. (1618-095X (Electronic)).

57. Kozikowski AP, Shen SA-O, Pardo M, Tavares MA-O, Szarics D, Benoy V, et al. Brain Penetrable Histone Deacetylase 6 Inhibitor SW-100 Ameliorates Memory and Learning Impairments in a Mouse Model of Fragile X Syndrome. (1948-7193 (Electronic)).

58. Hasan MR, Kim J-H, Kim YJ, Kwon KJ, Shin CY, Kim HY, et al. Effect of HDAC Inhibitors on Neuroprotection and Neurite Outgrowth in Primary Rat Cortical Neurons Following Ischemic Insult. Neurochemical Research. 2013;38(9):1921-34.

59. Dai C, Wang X, Liu R, Gao W, Zhang H, Yin Z, et al. ACY1215 Exerts Anti-inflammatory Effects by Inhibition of NF-kappaB and STAT3 Signaling Pathway to Repair Spinal Cord Injury. Biological and Pharmaceutical Bulletin. 2024;47(10):1734

EP - 45.

60. Tang P, Hou H, Zhang L, Lan X, Mao Z, Liu D, et al. Autophagy Reduces Neuronal Damage and Promotes Locomotor Recovery via Inhibition of Apoptosis After Spinal Cord Injury in Rats. Molecular Neurobiology. 2014;49(1):276-87.

61. Rahman M, Awosika AO, Nguyen H. Valproic Acid. BTI - StatPearls.

62. Villani SA-O, Dematteis G, Tapella LA-O, Gagliardi M, Lim DA-O, Corazzari MA-O, et al. Quantification of the Chemical Chaperone 4-Phenylbutyric Acid (4-PBA) in Cell Culture Media via LC-HRMS: Applications in Fields of Neurodegeneration and Cancer. LID - 10.3390/ph16020298 [doi] LID - 298. (1424-8247 (Print)).

63. Mann BS, Johnson Jr Fau - Cohen MH, Cohen Mh Fau - Justice R, Justice R Fau - Pazdur R, Pazdur R. FDA approval summary: vorinostat for treatment of advanced primary cutaneous T-cell lymphoma. (1083-7159 (Print)).
